# Climate and the Timing of Imported Cases as Determinants of the Dengue Outbreak in Guangzhou, 2014: Evidence from a Mathematical Model

**DOI:** 10.1371/journal.pntd.0004417

**Published:** 2016-02-10

**Authors:** Qu Cheng, Qinlong Jing, Robert C. Spear, John M. Marshall, Zhicong Yang, Peng Gong

**Affiliations:** 1 Ministry of Education Key Laboratory for Earth System Modeling, Center for Earth System Science, Tsinghua University, Beijing, People’s Republic of China; 2 Environmental Health Sciences, School of Public Health, University of California, Berkeley, Berkeley, California, United States of America; 3 Department of Infectious Diseases, Guangzhou Center for Disease Control and Prevention, Guangzhou, People’s Republic of China; 4 Department of Medical Statistics and Epidemiology, School of Public Health, Sun Yat-Sen University, Guangzhou, People’s Republic of China; 5 Divisions of Biostatistics and Epidemiology, School of Public Health, University of California, Berkeley, Berkeley, California, United States of America; 6 Joint Center for Global Change Studies, Beijing, People’s Republic of China; Santa Fe Institute, UNITED STATES

## Abstract

As the world’s fastest spreading vector-borne disease, dengue was estimated to infect more than 390 million people in 2010, a 30-fold increase in the past half century. Although considered to be a non-endemic country, mainland China had 55,114 reported dengue cases from 2005 to 2014, of which 47,056 occurred in 2014. Furthermore, 94% of the indigenous cases in this time period were reported in Guangdong Province, 83% of which were in Guangzhou City. In order to determine the possible determinants of the unprecedented outbreak in 2014, a population-based deterministic model was developed to describe dengue transmission dynamics in Guangzhou. Regional sensitivity analysis (RSA) was adopted to calibrate the model and entomological surveillance data was used to validate the mosquito submodel. Different scenarios were created to investigate the roles of the timing of an imported case, climate, vertical transmission from mosquitoes to their offspring, and intervention. The results suggested that an early imported case was the most important factor in determining the 2014 outbreak characteristics. Precipitation and temperature can also change the transmission dynamics. Extraordinary high precipitation in May and August, 2014 appears to have increased vector abundance. Considering the relatively small number of cases in 2013, the effect of vertical transmission was less important. The earlier and more frequent intervention in 2014 also appeared to be effective. If the intervention in 2014 was the same as that in 2013, the outbreak size may have been over an order of magnitude higher than the observed number of new cases in 2014.The early date of the first imported and locally transmitted case was largely responsible for the outbreak in 2014, but it was influenced by intervention, climate and vertical transmission. Early detection and response to imported cases in the spring and early summer is crucial to avoid large outbreaks in the future.

## Author Summary

Dengue has not been considered to be a major problem in China since it is recognized as an imported disease and only 8,058 cases were reported from 2005 to 2013. However, in 2014 alone, 47,056 new cases were reported. In this study, a mathematical model was developed to determine the possible cause of this outbreak. The most important parameters found to underlie the pattern of a small outbreak in 2013 and a much larger one in 2014 was the timing of the first imported and locally transmitted case. The importance of precipitation and temperature was also confirmed by the simulation results under different climate scenarios. The model also suggests that the earlier and more frequent control interventions in 2014 targeting immature mosquitoes, such as emptying water containers, and adult control, were effective in preventing larger outbreaks. Furthermore, more attention should be paid to imported cases occurring between March 1^st^ and July 1^st^ to prevent early and prolonged transmission. Without early detection and response, the final outbreak size might otherwise be an order of magnitude or more the size when the imported case occurred outside this time period.

## Introduction

Dengue is a febrile illness caused by the dengue virus which is further classified into 4 serotypes (DENV 1–4), and transmitted by *Aedes aegypti* and *Aedes albopictus* mosquitoes. Classically, dengue virus infection produces mild flu-like fevers but can also result in lethal dengue hemorrhagic fever (DHF) and dengue shock syndrome (DSS) when infected a second time with a different serotype [1]. According to the World Health Organization (WHO), dengue is the fastest growing vector-borne disease in the world with only one thousand cases reported in the 1950s to more than 90 million cases in the 2000s [2]. Estimated from a systematic literature search, there were 96 million apparent dengue infections globally in 2010; however, an additional estimated 294 million infections were asymptomatic [3].

Dengue is believed to be an imported disease in mainland China, and 55,114 cases were reported from 2005 to 2014. Approximately 94 percent of the indigenous cases that occurred in this period were reported in Guangdong Province, and 83 percent of these Guangdong cases were in Guangzhou City [4]. In 2014, an unprecedented dengue outbreak hit Guangzhou, with 37,341 new cases contributing to 94 percent of the new cases from 2005 to 2014 in Guangzhou. The annual new cases in Guangzhou were normally lower than 150 except for the 765 in 2006, 1,249 in 2013 and 37,341 in the 2014 outbreak.

Guangzhou differs significantly from other dengue transmission areas with *Ae*. *albopictus* as the sole vector rather than *Ae*. *aegypti* [5]. Unlike *Ae*. *aegypti*, *Ae*. *albopictus* adapts to the cold winter in temperate and subtropical areas by diapausing, which gives it the ability to expand to higher latitudes. Normally, the adults cannot survive the low temperature in winter, but they can produce diapause eggs when the temperature becomes lower and the day becomes shorter [6,7]. These diapause eggs will not hatch until the next spring, when the temperature and water condition become favorable again. Moreover, the vertical transmission of dengue virus in *Ae*. *albopictus* is more efficient [8], with approximately 0.5 to 2.9 percent of the eggs laid by infected mosquitoes being infected [8–10]. When the vertical infected diapause eggs develop to adults in the next spring, they have the ability to infect humans immediately without biting infected humans, even causing a significant outbreak if there were sufficient infected eggs in the past year. This pathway might allow dengue to become endemic in Guangzhou. The other possibility for dengue to be endemic is through overwintering infected adults, especially when global warming increases the temperature in the winter. However, the daily mean temperature from December to February of the 30-yr average (the coldest three months) was 14.8°C, and that of 2013 was 14.4°C. Thus the possibility for infected adults to live through the winter of 2013 is relatively low, considering that the temperature in the winter of 2013 was not abnormally high. Mathematical models suggest that vertical transmission can increase the endemic level of the vector population and human population significantly [11]. However, *Ae*. *albopictus* is less efficient in transmitting dengue virus. Typical explosive DHF epidemics have not been found in the places where *Ae*. *albopicus* predominates over *Ae*. *aegypti*, such as parts of China, the Seychelles Islands, La Reunion Island, the Maldive Islands, historically in Japan and most recently in Hawaii [12,13].

Another possible causal factor for the 2014 outbreak in Guangzhou was the abnormally high precipitation in May and August which provided more breeding sites and increased the environmental carrying capacity for *Ae*. *albopictus* [14]. A third possibility was the early starting date of the outbreak, with the earlier imported cases occurring in the late spring and early summer leading to the greater final size of the epidemic as a result of the lengthened infection season before the decrease of *Ae*. *albopictus* abundance in the winter [15]. A multivariate Poisson regression analysis of the Guangzhou outbreak data was recently published that showed the number of imported cases, minimum temperature with a one-month lag and cumulative precipitation with a three month lag predicted the outbreak in 2013 and 2014 [16]. Here we use a mathematical model rather than statistical model to further explore the factors underlying these outbreaks since the structure of our model is based on mechanistic factors controlling both mosquito population dynamics and the dynamics of viral transmission explicitly and, therefore, should allow greater confidence in making predictions in the presence of environmental change [17].

There were 99 dengue transmission models cited in literature from 1970 to July 2012, most based mainly on the Ross-Macdonald model of malaria transmission, the classical theoretical framework for modelling mosquito-borne diseases [18]. However, the core assumptions of some of these models differs from Ross-Macdonald model in various ways. Examples include those explicitly modelling the mosquito immature stage population dynamics [19,20], the temperature-dependent extrinsic incubation period (EIP) [21], vertical and mechanical transmission [11,22], spatial heterogeneity [15,23], control strategies [24,25], or multiple dengue virus serotypes [26]. Stochastic models [15] or agent-based models [27] have also been developed to simulate the transmission dynamics of dengue virus. To emphasize local characteristics, we included only immature stage population dynamics, vertical transmission, control strategies, and temperature-dependent adult mosquito mortality rate, biting rate and the EIP. Coinfection, multiple pathogen types, and temporary immunity were not considered here since dengue virus 1 (DENV-1) has been the only predominant serotype found in Guangzhou since the 1990s [4]. Though the test results for 2014 are not ready, in the 1,249 cases of 2013, 1,243 are DENV-1 cases and only 6 are dengue virus 2 (DENV-2) cases. Spatial distributions of the mosquitoes, and heterogeneous biting were not considered here mainly because of limited data availability.

In this paper, a population level deterministic mathematical model including explicitly modelled water level and mosquito population in different life stages was developed. Then the parameters in the model were estimated via successive cycles of fitting. Observed monthly mosquito index was used to validate the mosquito submodel. Finally, different scenarios were created to investigate the important mechanisms responsible for the unprecedented outbreak of dengue in 2014 in Guangzhou City.

## Methods

### Ethics statement

The study was reviewed and approved by the Ethics Committee of the Guangzhou Center for Disease Control and Prevention. All the patient data were de-identified and the data were analyzed anonymously.

### Study areas

**Fig 1:**
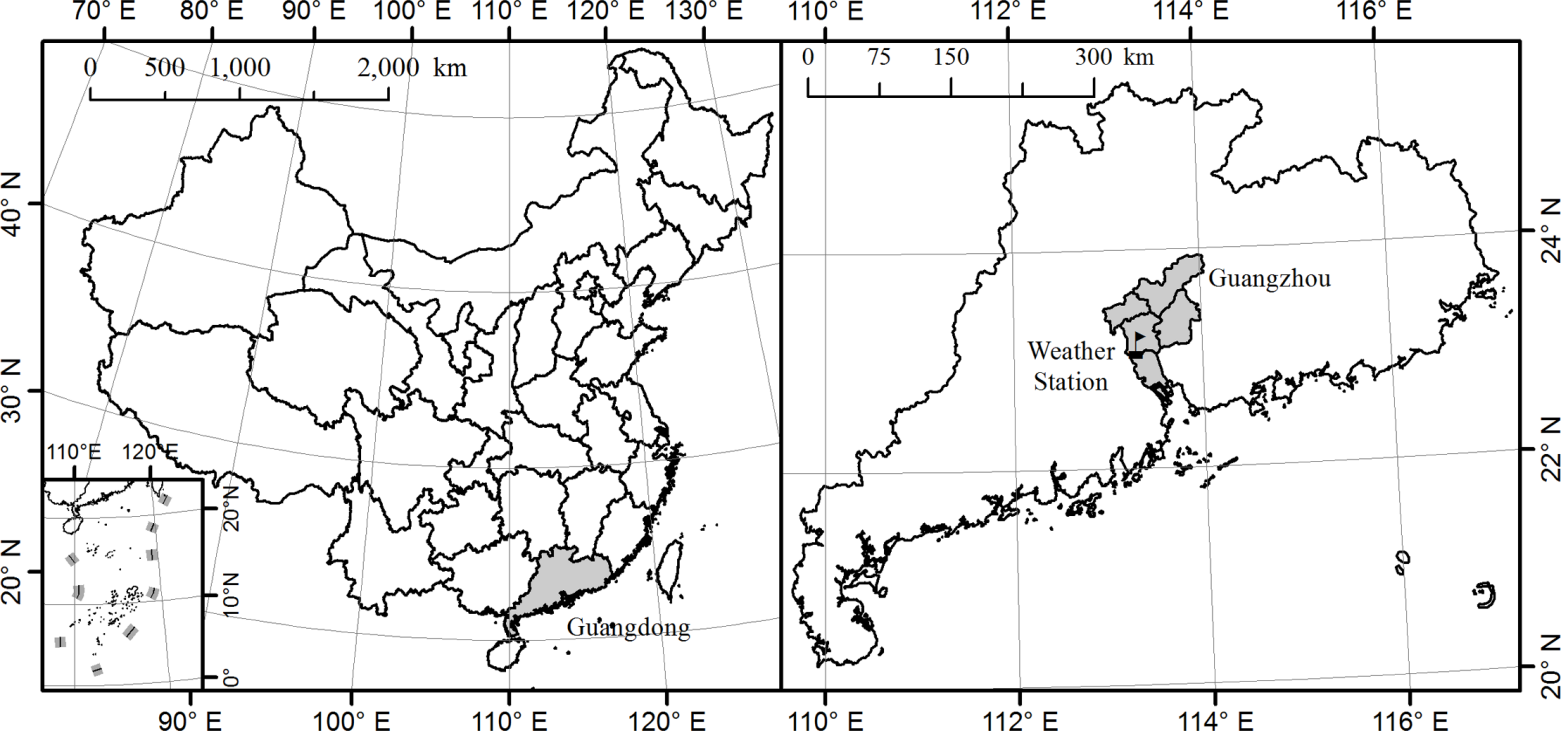
Location of study area, Guangzhou, in China.

Guangzhou is the capital and largest city of Guangdong Province, with a total area of 7,434 square kilometers [28] and a population of 13 million at the end of 2013 [29]. (Fig 1). It is one of the most urbanized areas and the center of China's economic growth. With the Tropic of Cancer crossing just north of the city, Guangzhou has a humid subtropical climate with hot and wet summers and mild and dry winters. The annual average temperature is approximately 21.5°C. January is the coldest month with an average temperature of 13.0°C while the hottest is July at 28.5°C. Annual rainfall varies from 1,612 to 1,909 mm, with more than 80 percent occurring between April and September [30]. The wet and warm climate is favorable for the growth of *Ae*. *albopictus*, which is the secondary vector for dengue virus in the world but the sole vector in Guangzhou [31,32]. Though dengue is not endemic in Guangzhou, more than 0.30 million travelers from dengue endemic countries such as Malaysia, Singapore, Indonesia, Thailand and India visit Guangzhou each year. These countries are also the top choices for outbound travelers from Guangzhou [33]. Since the natural and socio-economic conditions in Guangzhou are conducive to mosquito growth and reproduction, high densities of *Ae*. *Albopictus* together with dengue-infected travelers present a high potential for initiating local spread of the disease [31].

### Data collection

Dengue is a notifiable disease in China which means that, once diagnosed, cases must be reported to the web-based National Notifiable Infectious Disease Reporting Information System (NIDRIS) within 24 hours. All case reports used in this analysis were diagnosed according to the National Diagnostic Criteria for Dengue Fever (WS216-2008) published by the Chinese Ministry of Health [34]. In addition, active case detections was carried out through field investigations in the communities with confirmed dengue cases [14]. Cases were then divided into indigenous and imported cases based on whether the patient traveled to a dengue endemic area and was bitten by mosquitoes there within 15 days of the onset of illness [14]. A list of daily reported new cases for 2013 and 2014, obtained from Guangzhou Center for Disease Control and Prevention (Guangzhou CDC), was used to calibrate the model. This dataset was published online in the transmission season on the website of the Health Department of Guangdong Province (http://www.gdwst.gov.cn/). There were a total of 1,249 and 37,341 reported cases for 2013 and 2014, respectively.

Monthly mosquito surveillance reports consisting of the Breteau Index (BI) and the Mosquito Ovitrap Index (MOI) in 2013 and 2014 were also obtained from Guangzhou CDC and used to validate the mosquito submodel (S1 Table). BI is the number of positive containers with *Ae*. *albopictus* larva per 100 houses inspected, and is considered to be the best single index for *Aedes* density surveillance [14]. MOI is the percentage of *Ae*. *albopictus* positive ovitraps in all ovitraps collected from a specified area, and reflects the abundance of the adults [35].

Daily temperature, rainfall and evaporation data for Guangzhou from 2012 to 2014, which were used as inputs to the model, were downloaded from the China Meteorological Data Sharing Service System (CMDSSS) (http://cdc.nmic.cn/). In addition, climate data from 1985 to 2014 were also retrieved from CMDSSS to calculate 30-year daily average values.

Population data for the human submodel was obtained from the Guangdong Statistical Yearbook on China Infobank (http://www.bjinfobank.com/). This data was also used to estimate human birth rate and death rates in Guangzhou [36–38].

### Model description

**Fig 2:**
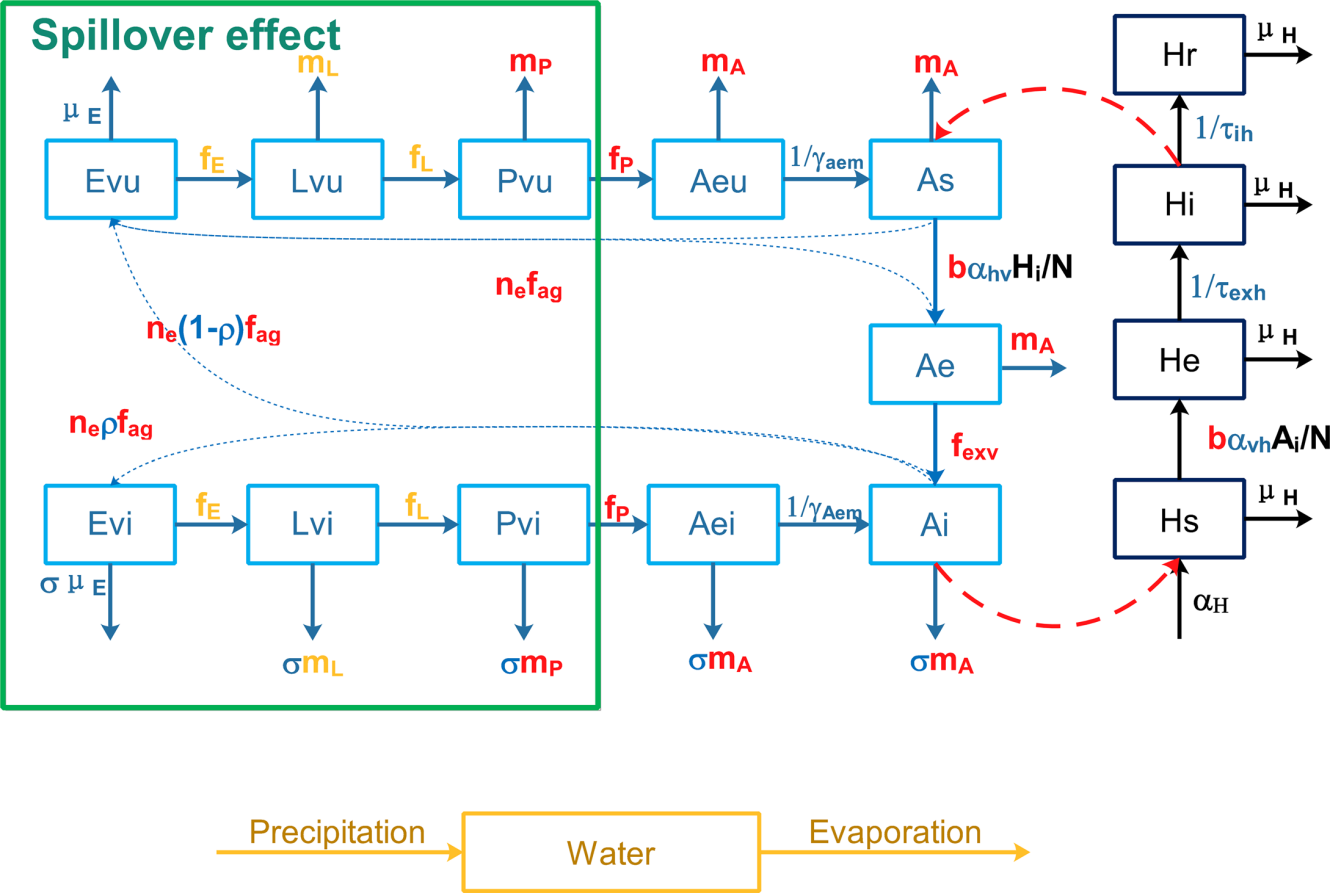
Flow chart for the transmission of dengue virus between mosquitoes and humans. Blue Greek letters indicate constant rates which need to be estimated and black letters for constant rates estimated from [36–38]. Yellow English letters indicate temperature and density-dependent functions. Red symbols indicate only temperature dependency and green rectangles on the left side indicate the state variables affected by the spillover effect.

A deterministic mathematical model was developed to interpret the transmission of dengue in Guangzhou city based on the Ross-Macdonald model [39,40], which is a basic framework widely used to study the dynamic transmission of mosquito-borne diseases. Fig 2 presents the structure of our model with Table 1 showing the definition for each symbol in this figure. Temperature can influence the development rate, death rate of immature mosquitoes, average duration of and number of eggs laid each gonotrophic cycle, biting rate and the EIP of dengue virus [41–43]. The form of temperature-dependent functions were based on [20,41], and the coefficients were estimated from experiments on *Ae*. *albopictus* strains from Guangzhou and adjacent areas [42,44]. Density of the larvae also plays an important role in the development rate of eggs and larvae, and the death rate of larvae. The form of density forcing rates were taken from [27]. More detailed information about the parameters, temperature or density forcing functions for *Ae*. *albopictus* development and death rates, and the differential equations for the model can be found in the S1 File.

**Table 1:** Description of parameters and notation in the model.

Parameter	Description	Typical values		Reference
*Constant rates*				
μ_H_	Mortality rate for residents in Guangzhou (day^-1^)	3.5×10^−5^	[36–38]	
α_H_	Population growth rate in Guangzhou (day^-1^)	8.1×10^−5^	[36–38]	
ξ	Sex ratio of *Ae*. *albopictus* at the emergence (dimensionless)	0.5	[45,46]	
*Parameters need to be estimated*				
μ_E_	Egg mortality rate (day^-1^)	0–0.1	[47]	
θ	The ratio of minimum egg hatching rate to ideal egg hatching rate (dimensionless)	0–1	To our best knowledge	
λ	The ratio of minimum larvae development rate to ideal larvae development rate (dimensionless)	0–1	To our best knowledge	
ω_0_	The maximum heavy rain washout fraction (dimensionless)	0–1	To our best knowledge	
ω_min_	Minimum water level (mm)	0-ω_max_	[27]	
ω_max_	Maximum water level (mm)	200–2000	[27]	
π_max_	Maxmium carrying capacity for immature stages (mosquito)	1.0×10^6^−1.2×10^7^	To our best knowledge	
γ_aem_	Duration from emerging adults to adults (day)	1–7	[42,46,48]	
μ_em_	Mortality during adult emergence (day^-1^)	0–0.2	[47]	
σ	The ratio of infected to uninfected immature and mature mosquito death rate (dimensionless)	1–3	[49]	
ρ	Vertical transmission rate, the proportion of infected eggs laid by infected mosquitoes (dimensionless)	0–0.2	[8–10]	
τ_exh_	Intrinsic incubation period (day)	4–8	[50]	
τ_ih_	Recovery period (day)	4–8	[51]	
α_vh_	Transmission probability from vector to human (dimensionless)	0–1	To our best knowledge	
α_hv_	Transmission probability from human to vector (dimensionless)	0–1	To our best knowledge	
φ	Reporting rate	0–1	1/3.2 in [52]	
β_2013_	Time for the imported case in 2013	521–571 (Jan 1st, 2012 as day 1) ^a^	Outbreak started on Day 561	
β_2014_	Time for the imported case in 2014	853–903 ^a^	Outbreak started on Day 893	
μ_a_	The survival rate for adults after intervention (day^-1^)	0–1	To our best knowledge	
μ_i_	The survival rate for immature stage after intervention (day^-1^)	0–1	To our best knowledge	
*Temperature-dependent rates (See mathematical expression in S1 File)*				
m_P_	Temperature-dependent mortality rate for pupa (day^-1^)	Function (S1 File)		
m_A_	Temperature forcing mortality rate for adult mosquitoes (day^-1^)	Function (S1 File)		
f_P_	Temperature forcing development rate for pupa (day^-1^)	Function (S1 File)		
f_exv_	1/Temperature-dependent EIP (day^-1^)	Function (S1 File)		
f_ag_	1/Temperature-dependent duration for gonotrophic cycle (day^-1^)	Function (S1 File)		
n_e_	Temperature-dependent eggs per gonotrohpic cycle (per female)	Function (S1 File)		
b	Temperature-dependent biting rate (day^-1^)	Function (S1 File)		
*Temperature- and density-dependent rates (See mathematical expression in S1 File)*				
m_L_	Temperature- and density-dependent mortality rate for larva (day^-1^)	Function (S1 File)		
f_E_	Temperature- and density-dependent egg development rate (day^-1^)	Function (S1 File)		
f_L_	Temperature- and density-dependent larvae development rate (day^-1^)	Function (S1 File)		

Some of the parameters do not occur in the flow chart, only in the equations, see S1 File for detailed information.

^a^ The range of timing of the first imported cases equals to the beginning time of local transmission – 15 days (Extrinsic incubation time + intrinsic incubation time in summer) ± 25 days.

The model includes several modifications to the Ross-Macdonald framework to incorporate the influence of climate factors, vertical transmission and local interventions. First, the immature aquatic phases of *Ae*. *albopictus* were modeled explicitly since the development rate of eggs, larva, and pupa, as well as the mortality of larva and pupa can be influenced by temperature and density. Second, a SEI (Susceptible, Exposed, and Infected) model was used for mosquito submodel instead of a SI (Susceptible and Infected) model to capture the temperature-dependent pathogen latency in *Ae*. *albopictus*. Thirdly, an element to reflect mosquitoes infected by vertical transmission was added, because *Ae*. *albopictus* has the ability to transmit dengue virus vertically through eggs, with a filial infection rates ranging from 0.5 to 2.9% for Dengue-1 virus [8]. Fourthly, we explicitly modeled the water availability by including evaporation, rainfall, and maximum and minimum water level (See details in S1 File). The environmental carrying capacity for mosquitoes will increase when the water level rises, and the density-dependent death rate will decrease in a short period. Furthermore, a spillover effect is triggered when there is an extreme rainfall event and the water level is close to the maximum water level, resulting in a loss of immature mosquitoes. The ideal death rate of larva and the development rate of eggs and larva depend only on temperature. However, the real death rate also depend on the water-level or density of the larva (See S1 File for more information). Similarly, the control intervention to empty water containers can also remove a fraction 1-μ_i_ of water and immature mosquitoes, while ultra-low-volume (ULV) aerosol applications of insecticides can kill a fraction 1-μ_a_ of adult mosquitoes. In addition, temperature-dependent biting rate and the number of eggs per gonotrophic cycle were incorporated to better represent the effects of climate on mosquito population dynamics. Since dengue is still considered as a non-endemic disease in China, which means new autochthonous cases occur only after imported cases, an imported case input was added to the system at day β_2013_ and β_2014_ (January 1^st^, 2012 as day 1) to initiate the outbreak in 2013 and 2014, respectively. Instead of using the date of the first reported imported case, we treated the timing of the first imported case as a parameter, since the outbreak may be started by an unreported or asymptomatic case. We only added the first imported case to the system and left out all the other subsequent imported cases, because it was a small number when compared with the number of infectious people after the rapid local transmission began, and was reasonable to be ignored. And because *Ae*. *albopictus* will survive adverse winter temperatures as diapausing eggs, the development rate from eggs to larva is assumed to be zero from late October to early March [53]. The reporting rate φ was also included to account for the asymptomatic and unreported dengue infections.

In summary, a SEI model was used for the vector submodel and SEIR (Susceptible, Exposed, Infected and Recovered) model for the human submodel (Fig 2). Five different life stages for mosquitoes were considered: three aquatic stages (E, eggs; L, larva; P, pupa), one emerging adult stage (Ae), and one biting and reproductive adult stage (A). Subscripts u and i were used to represent uninfected and vertical infected aquatic phases and emerging adults; while s, e, and i were used to denote the populations of susceptible, exposed, and infected adults. Analogously, the human population was divided into four subclasses: Hs, He, Hi, and Hr, which stands for susceptible, exposed, infected and recovered humans, respectively.

All the analyses were conducted in R 3.2.0 [54], and the differential equations in the model were solved by R package deSolve [55]. The model was run over the period 2012 to 2014, though the focus is on simulating the dengue outbreak in 2013 and 2014. The mosquito abundance for only the first simulated year is affected by the initial value for eggs, and the following years showed no memories from previous years [56], so an extra year was needed to achieve a stable mosquito population for 2013 and 2014. However, for simplicity, we assumed that there was no imported cases in 2012. The only possibility for dengue cases in 2012 to affect the next two years was through vertical transmission. Taking into account the low vertical transmission rate and the small number of dengue cases in 2012 (139 cases), we assumed that the influence of 2012 on the next two years was negligible.

### Model calibration

Typical of the class of mechanistic disease transmission models used here, there are a large number of parameters with substantial uncertainty in their values. In addition, in this analysis we place considerably more confidence in the timing and pattern of the field data describing human cases and mosquito infection and abundance in Guangzhou in 2013 and 2014 than in the precise numbers reported on any day. As a result, we chose to address the issue of parameter estimation using a strategy that has been called regional sensitivity analysis or RSA [57]. This approach begins with the specification of a region of parameter space thought to include the range of feasible values of each parameter with high probability (As the typical value for each parameter in Table 1). Monte Carlo simulation runs are then conducted to assess the performance of the model over this parameter space. Here we define this space by specifying the univariate marginal distributions of the model parameters need to be estimated, as given in Table 1, each of which we assume to be independent.

**Fig 3:**
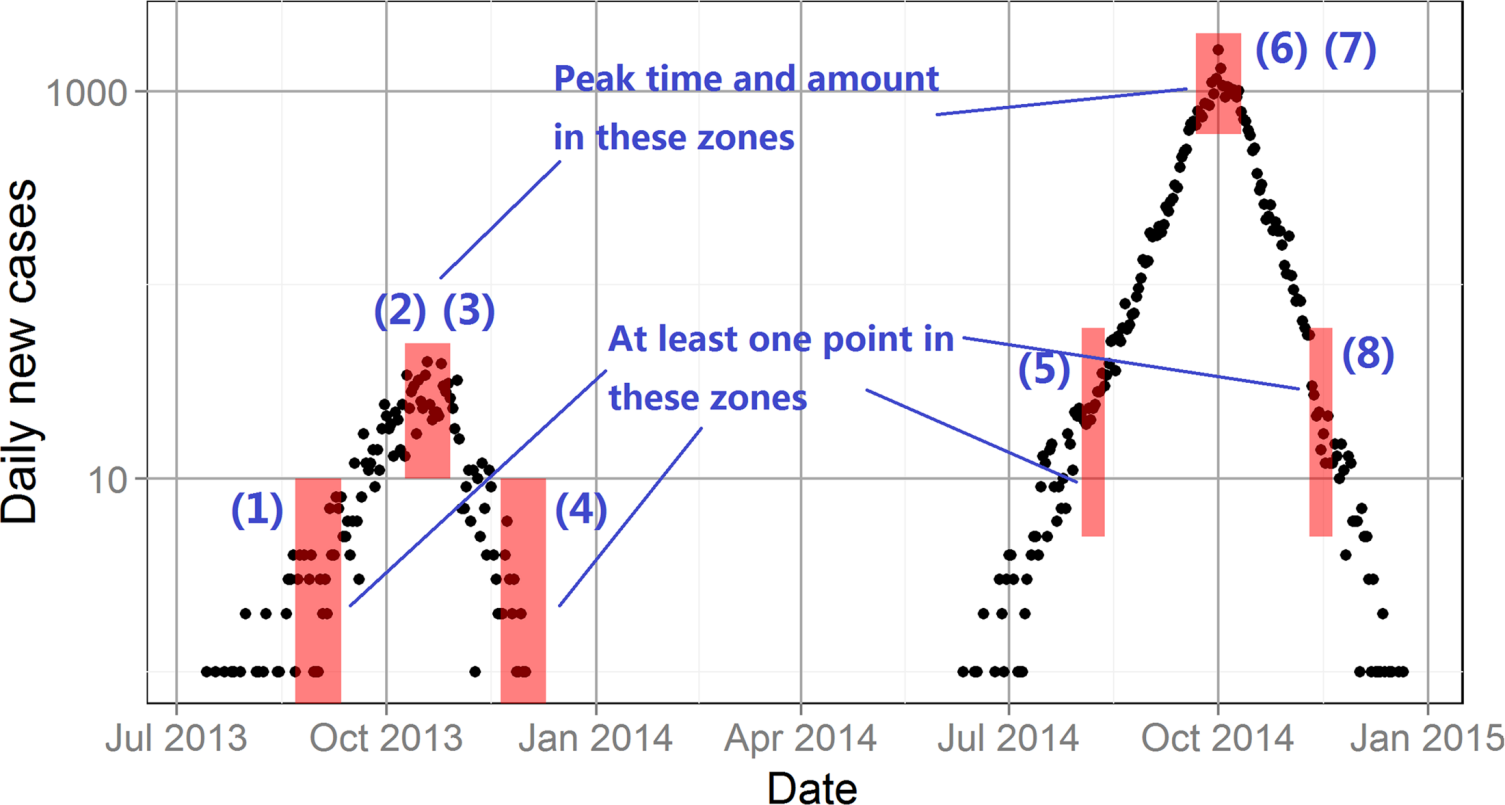
The epidemic curve and passing criteria for Guangzhou Dengue outbreaks in 2013 and 2014. Black dots represent the amount of daily new cases, and the red shaded rectangles show the time and amount window for the eight criteria (See the detailed descriptions for these criteria in S1 File).

Classification criteria are then defined and applied to the output of the model to determine if a particular realization captures the essential features of the pattern of daily case reports. Fig 3 shows the specific criteria for the 2013 and 2014 Guangzhou dengue case reports (See the detailed criteria in S1 File). If a particular model run results in a case report trajectory passing through all six of the shaded windows, the model is classified as a “pass”, that is as having adequately mimicked the pattern of the field data used for calibration.

Passing and failing parameter vectors are then collected for subsequent analysis. Usually the first simulation experiments using the RSA approach result in a very small fraction of passes and these vectors extend over almost the entire range of any of the univariate prior distributions. This is a result of the fact that there are many parameter combinations that can produce the same patterns of model response and their correlation structure is usually very complex in the high dimensional parameter space being sampled. Non-uniqueness of model parameterization of this sort is an issue about which there is a substantial literature particularly in the field of hydrology [57]. In the present case, 74 passing parameter vectors were obtained in 410,594 initial simulation runs which we term Cycle 1. In the S2 File the sample cumulative distribution functions are shown for each parameter for passes and fails. As shown there, some parameter distributions that differ little between passes and fails which gives little clue as to parts of the range of that parameter where passes are more likely. The value of d_m,n_, the Kolomogorov statistic, is a measure of the maximum difference between the two distributions and can be used as a rough index of sensitivity. Very large differences can be seen for some parameters in Cycle 1, for example β_2013_, μ_i_, μ_em_ and ω_max_.

In view of the very low pass rate of the first set of simulations, we chose to use the outcome of the Cycle 1 experiments to seek a subspace in which passing parameters were more likely to be found. This was done by trimming the ranges of parameters with large values of d_m,n_. Trimming was an ad hoc procedure based on trimming the range of parameters with few passes at either the high or low end of the sample distribution function of passes. A total of 4 trimming cycles were conducted resulting in a pass rate of 3.19% in the final subspace, Space 5, an increase of about 175 times over the initial space, Space 1. The marginal distributions of the parameters in Space 5 now show much reduced differences between passing and failing distributions as discussed below. We regard a highly trimmed parameter range and a large decrease in d_m,n_ between spaces 1 and 5 as evidence of the importance of a parameter in producing simulations meeting the pass criteria. However, we note that a parameter may be very important, but if initial uncertainty in its value is small, that is the prior range is narrow, there may be little difference in the marginal distributions under passes versus fails. A second situation in which a parameter can show little difference in its pass/fail marginal distributions yet be important can occur if there are interactions with other parameters not reflected in the marginal distributions. The pairwise correlation matrix can give some clues to such situations and will be discussed below for Space 1 and Space 5. (See S2 Table for the pairwise correlation matrices for parameter values Cycle 1 and Cycle5)

**Fig 4:**
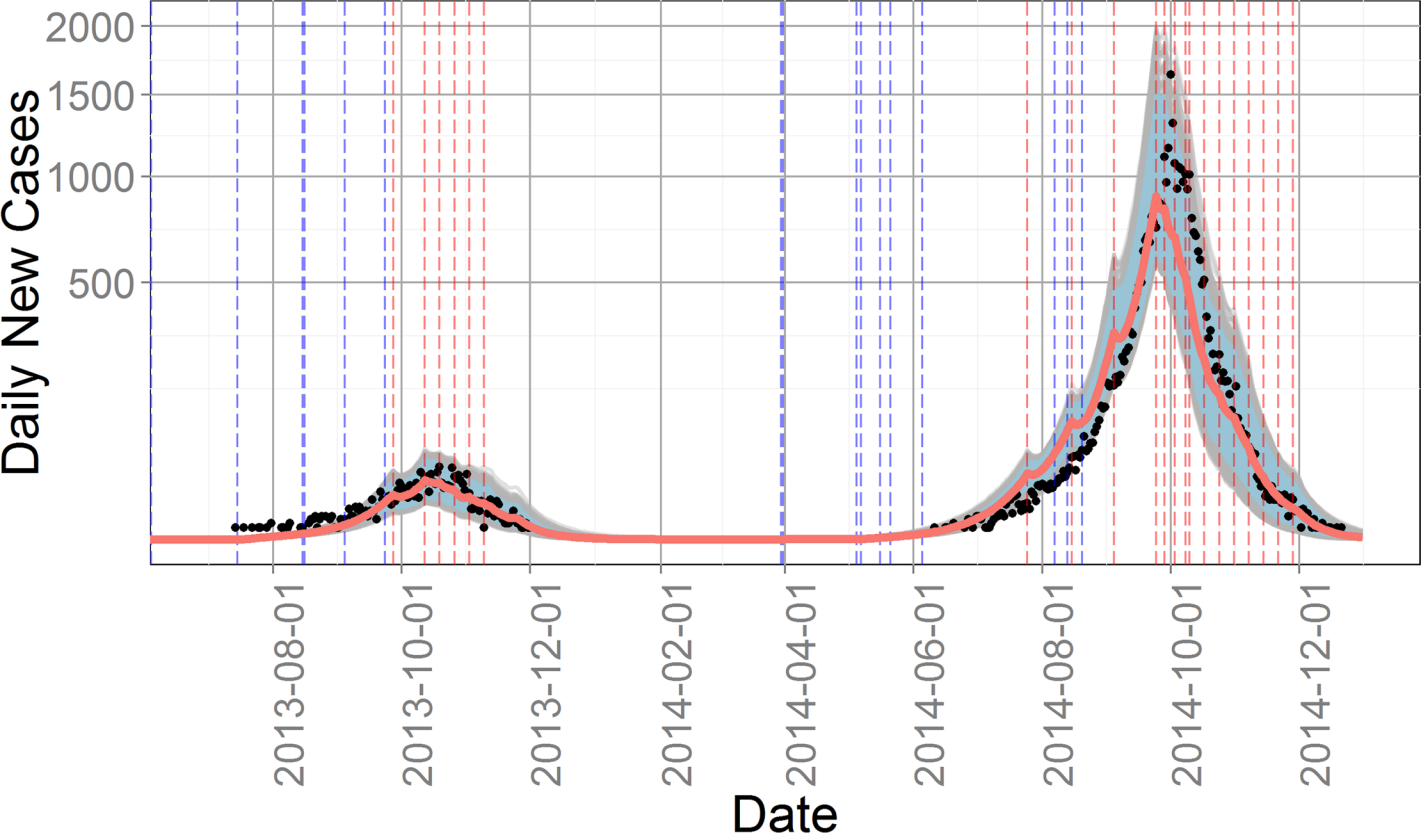
Trajectories for daily new cases of the 637 passing parameter sets in Cycle 5. Black dots indicate the number of daily new cases from Guangzhou CDC, while gray lines are model outputs and red line is the median for all outputs. Blue and red vertical dash lines stand for washout and intervention days, respectively. Blue shaded area for the 90 percent interval for all 637 simulations.

Fig 4 summarizes the case report data for 2013 and 2014 and shows the envelope of 637 passing simulation trajectories from the Space 5 parameter distributions. The daily number of new cases output by the model was calculated as the number of individuals entering the compartment Hi times the reporting rate φ. The median final epidemic size for 2013 and 2014 was 1,044 and 30,863, respectively, for the 637 passing parameter sets of Cycle 5. Although the envelope of passing simulations contains the observed peak values in both years, the median passing peaks were 16% and 17% lower than the observed peaks respectively.

**Fig 5:**
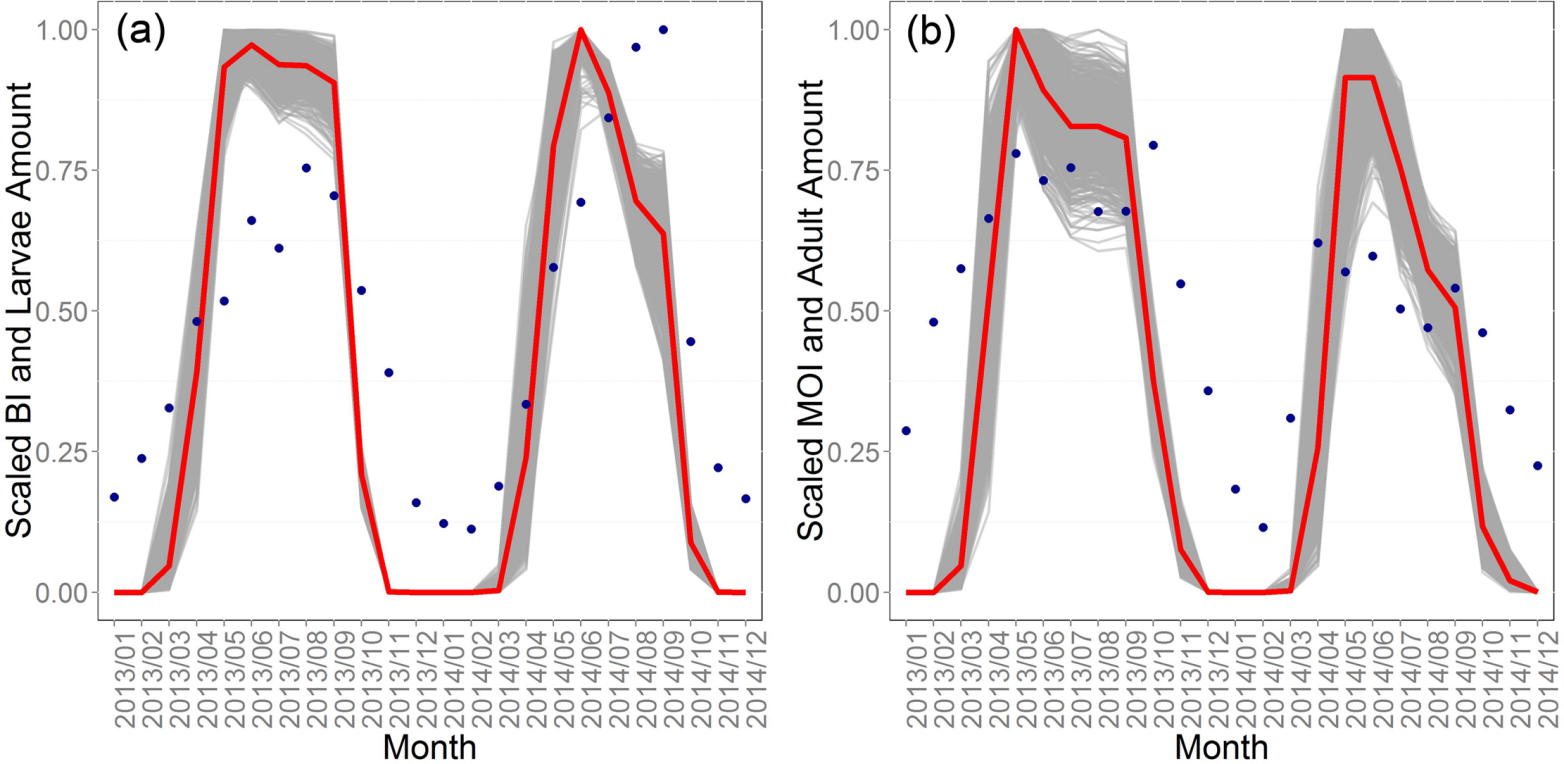
Mosquito submodel patterns. The scaled 637 simulated results and field data for (a) larva and (b) adults. Gray lines show model output, red lines median output, and dark blue points show mosquito surveillance data acquired from Guangzhou CDC.

Fig 5 shows the Cycle 5 simulation results and field data for larvae and adult mosquitoes. We aggregated the monthly average amount of larva and adults from daily model output of the 637 passing simulations, then normalized them to 0 to 1 and plotted them against the normalized BI and MOI data from Guangzhou CDC. Mosquito surveillance data in 2012 was not used in the validation, because the mosquito abundance in the first simulated year can be affected by the initial value for eggs. Entomological surveillance data recorded only the absence/presence not the number of *Ae*. *albopictus* in each container, so it is only a proxy of the abundance. The minimum, maximum, mean and standard deviation for Pearson’s correlation between scaled model output larva amount and BI were 0.76, 0.86, 0.82, and 0.02, respectively. And the correlation for scaled model output adult amount and MOI ranged from 0.65 to 0.80, with a mean of 0.74 and standard deviation of 0.03. The BI and MOI data were not used in calibration but the patterns produced by the model, as shown in Fig 5, confirm that the model is producing realistic patterns of mosquito abundance over time.

Table 2 shows the Space 1 versus Space 5 marginal distribution comparisons with RR (range reduction) denoting the fractional reduction of the range in each parameter. The ranges of six parameters were unaltered and nine were reduced by 50% or more of their initial range. The nine fall into three types of parameters, those associated with vector population dynamics and infection (μ_E_, λ, σ, and α_hv_), those related to the timing and reporting of imported cases (β_2013_, β_2014_, and φ), and those associated with the effectiveness of mosquito control interventions (μ_a_ and μ_i_).

**Table 2:** The range and d_m,n_ for each parameter in Cycle 1 and Cycle 5, and the RR from Cycle 1 to Cycle 5.

Parameter	Cycle 1		Cycle 5		RR (%)
	Range	d_m,n_	Range	d_m,n_	
μ_E_	0–0.1	0.23	0.02–0.05	0.11	70
θ	0–1	0.20	0.4–1	0.06	40
λ	0–1	0.26	0.4–0.8	0.03	60
ω_0_	0–1	0.10	0–1	0.06	0
ω_min_	0 – ω_max_	0.19	0 – ω_max_	0.03	0
ω_max_	200–2000	0.31	250–1400	0.07	36
π_max_	1×10^6^–1.2×10^7^	0.15	2×10^6^–9×10^7^	0.11	36
γ_aem_	1–7	0.10	1–7	0.02	0
μ_em_	0–0.2	0.13	0.08–0.2	0.03	40
σ	1–3	0.32	1.8–2.7	0.09	55
ρ	0–0.2	0.11	0–0.2	0.05	0
τ_exh_	3–9	0.15	3–9	0.07	0
τ_ih_	3–9	0.06	3–9	0.04	0
α_vh_	0–1	0.11	0.2–0.9	0.06	30
α_hv_	0–1	0.12	0.2–0.6	0.12	60
φ	0–1	0.10	0.1–0.5	0.08	60
β_2013_	520–570	0.17	560–570	0.07	80
β_2014_	853–903	0.52	850–870	0.06	60
μ_a_	0–1	0.22	0.4–0.6	0.04	80
μ_i_	0–1	0.28	0.65–0.9	0.05	75

The pairwise correlation matrices for the passing parameter distributions in Space 1 and Space 5 are shown in S2 Table. For Space 1, the same 9 parameters with large range reductions show high correlations with one or more of others in that group. There is also a very high correlation between ω_min_ and ω_max_, an artifact that is imposed by the model structure. In the Space 5 correlations, all of the high values from Space 1 are lower, and most very much lower, as might be expected. However, some new correlations emerge, notably with π_max_, the maximum carrying capacity for immature stages of the mosquito. These correlations are with μ_em_, mortality during adult emergence, and the human to vector, α_hv_, and vector to human, α_vh_, transmission probabilities.

We do not believe we have access to additional field data or other information which will allow significant further reduction in Space 5 or point to other areas in the parameter space that might suggest alternative underlying processes to be driving the observed patterns of behavior of the system. Hence, parameter vectors meeting the passing criteria sampled from Space 5 will be used in the subsequent explorations of the key processes underlying the 2014 epidemic.

### Simulation experimental results

The year 2013 differed from 2014 in several aspects, notably the date of imported cases (β_2013_ and β_2014_), climate, the time and frequency of the interventions, and the number of eggs infected by vertical transmission from the previous year.

**Fig 6:**
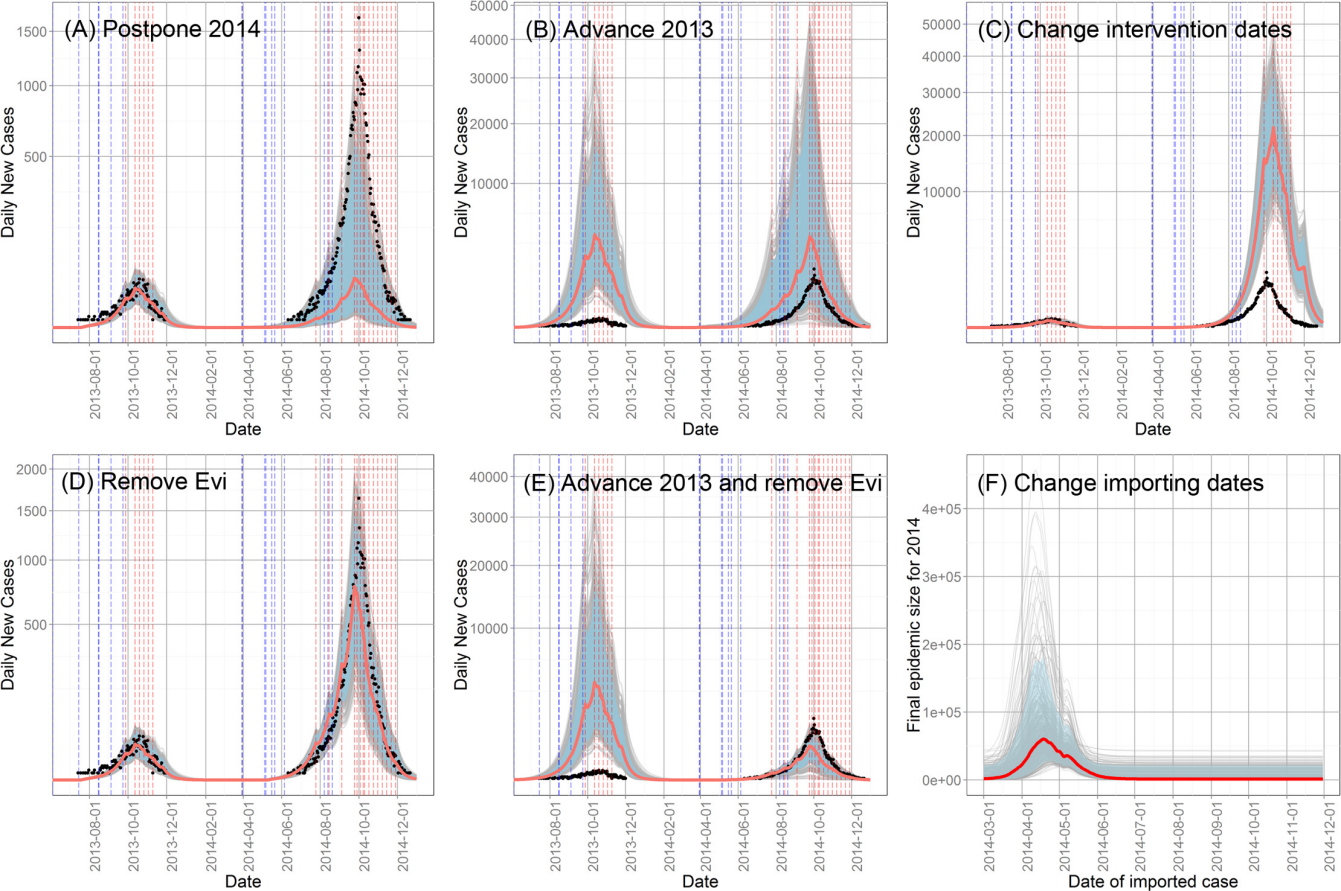
Trajectories of daily new cases under different scenarios. (A) Postponing the date of imported case in 2014; (B) advancing the date of imported case in 2013; (C) setting the intervention in 2014 to the same as that in 2013; (D) removing all the infected eggs at the beginning of 2014; (E) advancing the date of imported case in 2013 and removing all the infected eggs at the beginning of 2014; and (F) trajectories of the final epidemic size for 2014 after changing the date of imported case between March 1^st^ and November 30^th^. Black dots indicate for the daily reported case in 2013 and 2014. Gray lines indicate the trajectories for each simulation. Red lines indicate for the median and blue shaded area for the 90 percent interval for all 637 simulations.

We first explore the timing of imported cases, which is not the real timing of the first imported case reported to the NIDRIS, but a parameter we need to estimate, denoting the first imported case that starts the local transmission. The outbreak in 2014 started at June 11^th^, and peaked around October 1^st^, with a time interval of 112 days, while the smaller outbreak in 2013 began at July 14^th^, and peaked around October 19^th^, with an interval of 97 days. If the force of infection was the same for these two years, the final size of epidemic in 2014 would be significantly higher than that in 2013 on this basis alone. Without interventions, the peak occurs when the temperature drops to cause a sufficient combination of a decrease in biting rate and an increase in the mosquito death rate. Appropriately timed interventions reduce the abundance of mosquitoes, thus reducing the force of infection which results in an earlier peak. To make the two years comparable, we changed the date of the first imported case in 2014, β_2014,_ in the 637 passing parameter sets to β_2014_ + (Peak time 2013—β_2013_) (Scenario Postpone 2014). By doing this, we made the time interval between the imported case and the peak in 2014 equal to that in 2013. The case report trajectory for each run was recorded (Fig 6A). Then in another scenario (Scenario Advance 2013), we changed the β_2013_ to β_2013_ –(Peak time 2014—β_2014_), to attempt to produce an outbreak in 2013 with a size similar to the observed number in 2014 (Fig 6B). Furthermore, to investigate the relationship between the date of imported cases and the final epidemic size further, we kept all the other parameters the same as in the 637 passing sets, and only changed β_2014_ in each set to integers between Day 791 and 1066, that is from March 1^st^, 2014 to November 30^th^, 2014 (Scenario Change importing dates), and recorded the final epidemic size for each run (Fig 6F).

The results of these experiments are shown in Fig 6. When the time interval between the imported case and the peak in 2014 was changed to match that in 2013, only 30 parameter sets (4.7% of the original 637) mimicked the pattern of the outbreak in both years. The median final epidemic size of 2014 dropped to 1,474, similar to that of 2013 (Fig 6A). And when the time interval between the imported case and the peak in 2013 was increased to the same as that in 2014, none of the 637 parameter sets produced passing behaviors. As shown in Fig 6B, after the change, the peak number of cases was significantly higher in both years, with new median final outbreak sizes of 158,889, and 137,003 for 2013 and 2014, respectively. In summary, postponing the date of the import case in 2014 produces an outbreak whose scale is similar to that of 2013, and advancing the date of the import case in 2013 produces an outbreak even worse than observed in 2014. In addition, since all other parameters were unchanged except for β_2013_, the larger than observed outbreak in 2014 is attributable to vertical transmission, that being the only way that the situation in 2013 can influence that in 2014. A separate scenario was created, still by advancing β_2013_, but removing all infected eggs in the system at the beginning of 2014, as discussed below.

The final experiment on the timing of imported cases involved holding all parameters the same as in the 637 passes, except that of the date of imported case which was varied from March 1^st^ to November 30^th^. The final epidemic size for each run is plotted in Fig 6F and shows that when the first imported case occurs on April 18^th^, the median final epidemic size was the highest at 60,158. The final epidemic size became stable after July 1^st^ at approximately 1,350, similar to the observed size in 2013. These experiments clearly suggest that the date of the first imported cases was a crucial determinant of the severity of the 2014 epidemic.

**Fig 7:**
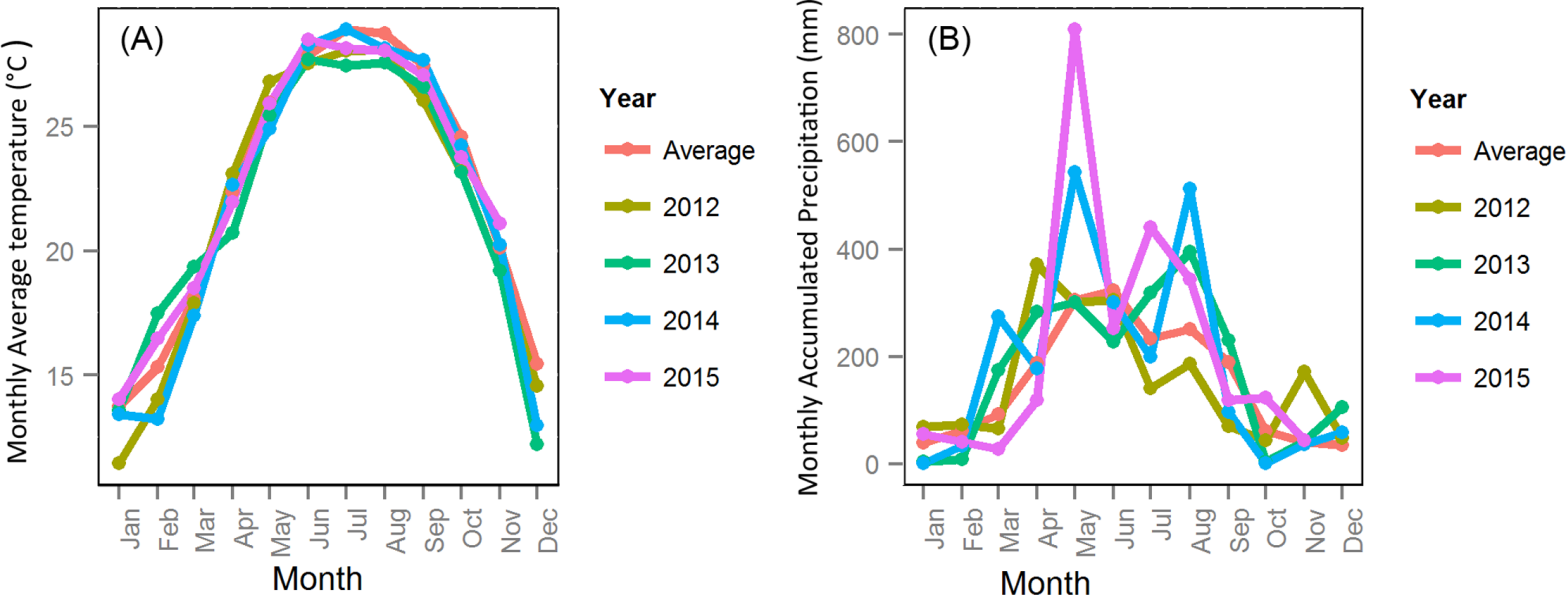
(A) Monthly average temperature; and (B) monthly accumulated precipitation for Guangzhou in 2012 to 2015 and the 30-year average.

However, the force of infection was not the same in 2013 and 2014. It is affected by mosquito abundance, biting rate, transmission probability from vector to human and transmission probability from human to vector. Though we assumed the transmission probabilities were the same for 2013 and 2014 (α_hv_ and α_vh_), the biting rate depends on temperature and the mosquito abundance depends on both temperature and precipitation. Hence, different scenarios were created to study the role of climate of the variations in climate depicted in Fig 7. Experiments were conducted in which the precipitation, temperature, and evaporation data of 2014 were replaced by data of 2012, 2013 or of the 30-yr average. Since the temperature in 2014 did not differ significantly from that in other years, while the precipitation in May and August 2014 were much higher, we also ran simulations with actual temperature and evaporation in 2014, but scaled the precipitation to 30 year average.

The new case trajectory for the real climate data was treated as baseline here, so the passing rate was 100 percent for the 637 parameter sets. Table 3 shows the results of the various experiments. As can be seen, the passing rate was relatively low, at only about 28 percent, when 30 year average precipitation was used to replace the real 2014 data (Scenario 2, 5, 6, 7, 8, and 13). Furthermore, the median peak size and final epidemic size were significantly lower than baseline. When the 2014 precipitation was used (Scenario 3, 9, 10, 11 and 12), the passing rate was around 65 percent but it was more than 80 percent when the 2013 or 2014 temperature was used. The median peak outbreak size was higher than baseline when 2014’s precipitation and average temperature were combined together (Scenario 3 and 11). The maximum difference between precipitation in 2014 and the 30-year average occurred in May and August, so we scaled the precipitation in these two months to their 30-year average. The passing rates were 65.0, 61.2 and 35.8 percent when we scaled only May, only August and both, respectively (Scenario 16, 17, and 18). When comparing Scenario 19, 20, 21 with 13, higher passing rate and average outbreak size are observed as a result of increasing the rainfall in May and August above the 30-year average. Rainfall in August seems to be slightly more important.

All the results suggest that the precipitation in 2014 played an important role in forming the outbreak, especially rainfall in May and August. However, the temperature in 2014 was lower than average in the spring and winter months, thus acting as a protective factor. That is, if the temperature in 2014 had been higher, the average outbreak size would have been higher as well.

**Table 3:** Passing rates, median peak sizes, and median final epidemic sizes under different climate scenario for 2014.

Scenario	Precipitation	Temperature	Evaporation	Passing rate	Median peak size	Median outbreak size
0	2014	2014	2014	100	894	30863
1	2013	2013	2013	42.7	549	18543
2	Avg	Avg	Avg	27.0	463	16842
3	2014	Avg	Avg	67.8	1010	34826
4	2013	Avg	Avg	57.8	756	26809
5	Avg	2014	Avg	26.5	369	13509
6	Avg	2013	Avg	22.6	342	11833
7	Avg	Avg	2014	31.4	538	18925
8	Avg	Avg	2013	26.7	430	16060
9	2014	2014	Avg	85.1	808	28074
10	2014	2014	2013	82.1	789	27127
11	2014	Avg	2014	64.2	1132	38879
12	2014	2013	2014	84.3	817	26493
13	Avg	2014	2014	30.5	424	15559
14	2013	2014	2014	60.8	679	23676
15	2012	2014	2014	35.6	518	18559
16	2014 May*0.56	2014	2014	65.0	690	23924
17	2014 August*0.49	2014	2014	61.2	666	23938
18	2014 May*0.56, August*0.49	2014	2014	35.8	504	18518
19	Avg May*1.79	2014	2014	36.4	513	18397
20	Avg August*2.04	2014	2014	40.2	538	18956
21	Avg May*1.79, August*2.04	2014	2014	54.0	649	22453
22	2013 May*1.81	2014	2014	77.2	763	26716

Avg, 30-yr average precipitation, temperature or evaporation.

The peak time of daily new cases is clearly sensitive to the date of interventions and the simulation results suggests that the interventions are very effective. The most common interventions in Guangzhou were emptying water containers and ULV spraying of adulticide, both conducted at neighborhood level and organized by neighborhood committee. Emptying water containers reduces the abundance of the immature stage, water level and environmental carrying capacity, thereby reducing adult abundance. ULV spraying of insecticide decreases the abundance of adults almost instantly. With a reduced vector to human ratio, the force of infection decreases while the recovery rate remains the same resulting in an earlier peak. The interventions in 2013 took place every Friday from October 9^th^ to November 10^th^, while in 2014 on every Friday from September 24^th^ to November 30^th^, as well as on July 25^th^, August 15^th^, September 4^th^ and 28^th^, and October 8^th^.

To determine the effectiveness of these interventions, we set interventions in 2014 the same as those in 2013 and recorded the trajectories (Scenario Change intervention dates). The intervention in 2013 took place later and has a much lower repetition frequency. No passes occurred after the changes, because the median peak size and overall outbreak size increased drastically to 21,808 and 843,430 respectively (Fig 6C), approximately 27 times the baseline value and 23 times the actual reported cases in 2014. The new peak time was October 12^th^, almost 15 days later than the observed peak, which again shows the importance of the time interval between the imported case and the peak.

In addition, the filial infection rate can also change the characteristic of an outbreak. To investigate the importance of vertical transmission, the number of infected eggs (Evi) was set to zero at the beginning of 2014 (Scenario Remove Evi), because this is the only way that the epidemic in 2013 can influence that in 2014. This change resulted in 490 passing simulations out of 637 runs. The median of peak size and outbreak size were 778 and 2,792, respectively, only slightly lower than the baseline (Fig 6D). In addition, when we investigated the role of timing of the imported case and changed β_2013_ to make the time interval between the import case and peak in 2013 the same as that in 2014, we found that the peak size and outbreak size in 2014 also increased and attributed this to vertical transmission. A scenario was also run with both an advanced import case date in 2013 and no infected eggs carried over from 2013 to 2014 (Scenario Advance 2013 and remove Evi). The peak and outbreak size dropped to 539 and 15,526, respectively, which suggested that the effect of vertical transmission should not be neglected when the outbreak size in the previous year was large (Fig 6E).

## Discussion

From our analyses, four factors appear to have been principally responsible for the pattern of the moderate outbreak in 2013 and the much larger one in 2014, namely the date of the first imported case, unusually high precipitation in 2014, interventions, and vertical transmission. We found the timing of first imported and transmitting case was the dominant feature responsible for this pattern. Furthermore, once the timing of imported case is fixed, climate significantly affects the dengue transmission dynamics. For example, precipitation in May and August, 2014 were found to have a moderate effect on the size of the outbreak, while temperature in 2014 was less favorable for the outbreak and suggests that if the temperature had been higher in the spring and winter months in 2014, the final outbreak size would have been even greater. Vertical transmission played a minor role in forming the pattern, but it is likely to be significant only when the outbreak size in the previous year is large. In addition, we found that the earlier and more frequent interventions in 2014 proved to be effective, otherwise the outbreak size might have been over an order of magnitude higher than the observed value.

The date of imported case was crucial in producing the outbreak pattern in 2013 and 2014. The date of the first imported case in our analysis is not the exact date of the first imported case, but a dummy variable indicating the time of the imported cases which starts the local outbreak. Since we have no information about which imported case will cause local transmission, the time of imported cases was set to be a parameter to be fitted in the model. Though imported cases occurred in almost every month, indigenous cases were mainly reported from July to November when the mosquito abundance and biting rate are higher, and the EIP is shorter. [4] Temperature and arrival date of the first infectious human also interact since early arrival will occur at lower temperature, but there is a longer time for transmission to increase before the beginning of winter season and thereby produce a larger outbreak [15]. That is, low temperature can increase the EIP as well as reduce the biting and the mortality rate resulting in fewer mosquitoes surviving to be infectious as was also shown in Fig 6F. Considering the tradeoff between higher biting rate and longer transmission season, a case imported around mid-April appears to have triggered the biggest outbreak in 2014. (Fig 6F) In addition, the number of imported cases also matters to the outbreak size [16]. However, we did not take this into account, since in our deterministic model one imported case is sufficient to initiate internal transmission.

Precipitation too can have both beneficial and detrimental effects on the abundance of *Ae*. *albopictus* and dengue transmission. *Ae*. *albopictus* mainly breed in flower pot trays, bamboo tubes, used tyres, disposable containers and surface accumulated water. Precipitation can change the water level in these containers and thereby affect the density-dependent development and death rate [27]. When the water level is higher, the environmental carrying capacity also increases; hence, the maximum number of mosquitoes the environment can support will also increase. Higher water level will also bring down the death rate and increase the development rate, so the survival rate of mosquitoes increases during such periods, and development from larvae to adult will be faster. On the other hand, heavy rainfall also can destroy breeding sites. When a heavy rain occurs at the time the water level is close to the maximum, some of the immature stage mosquitoes will be washed out of their containers (Spillover effect in Fig 2) making container habitats significantly less attractive to ovipositing females. Both mechanisms can cause population loss of *Ae*. *albopictus* [58].

In contrast, a study in France suggested that the heavy rainfall events can increase the risk of chikungunya [59]. In fact, the relationship between precipitation and mosquito abundance is complicated. We increased or decreased different amounts of rainfall in 10-day time windows, and ran the model with these new precipitation profiles. The result showed that the relationship between the amount of precipitation and mosquito abundance or the number of dengue cases was nonlinear, and there was no simple rule to predict the effects of rainfall or heavy rainfall.

According to Table 3, the temperature in 2014 was not as important as precipitation in causing the outbreak pattern because the inter-annual change of temperature is much smaller than that of precipitation. However, temperature plays an important role in controlling various aspects of the seasonal population dynamics of *Ae*. *Albopictus* as discussed above.

The vertical transmission rate was less important in determining the outbreak pattern in 2013 and 2014, though experiments have confirmed that adults hatched from infected diapause eggs can transmit dengue virus [60]. Our analysis suggested that with the small number of cases in 2013, it is impossible that the big outbreak size in 2014 was caused by only by vertical transmission, therefore dengue was still imported, not endemic, for the 2014 outbreak, which was also recognized by analyzing seasonality and virus source of dengue cases [4]. The probable sources of dengue virus detected in Guangzhou were mainly Thailand, Philippines, Indonesia, Vietnam, Cambodia, and Malaysia [14,61], all of which are also popular tourist destinations for residents in Guangzhou [33]. However, the influence of vertical transmission should not be neglected if a big outbreak occurred in the previous year. Considering the large amount of infected eggs left over from 2014 to 2015, the effect of vertical transmission in 2015 should be large, even can start a local outbreak without any imported case. However, there is no big outbreak in 2015, with 44 imported cases but only 57 indigenous cases, though the precipitation in May was higher and there are more imported cases than 2014. This is likely to be attributable to the extensive interventions in 2015. After the unprecedented outbreak in 2014, the government paid more attention to early detection of imported cases, early mosquito control (started in April compare with in the end of July, 2014), and the quarantine of every suspicious case. Moreover, residents in Guangzhou have more knowledge about the difference between dengue and influenza after 2014, so they are more likely to go to the hospital when symptoms occur and will be put quarantined immediately after confirmed, which can also reduce local transmission. However, due to the deterministic nature of this model, its use is only appropriate when the scale of the outbreak was big enough to ignore the stochastic effects, but the outbreak size in 2015 was relatively small, therefore we did not simulate the situation in 2015 here.

Currently, there is no effective commercial dengue virus vaccine available. Thus, the prevention of a dengue outbreak relies heavily on vector control. Container emptying and ULV spraying are the most common control strategies in China. Other approaches such as releasing *Wolbachia* infected male *Ae*. *albopicus* and introducing mosquito larvae-eating fish have also been adopted, though to a much smaller extent. Although the efficiency of ULV spray in controlling adult *Ae*. *albopictus* has been questioned over the years [24,62], larval source reduction has proven to be successful [62]. Since Guangzhou applied both strategies at the same time, we could not separate them. In addition, though it was argued that mosquito control strategies were often implemented after the peak of transmission and had little or no impact on dengue transmission [62], the first intervention in Guangzhou was timely, two months earlier than the peak, and does appear to have reduced the final epidemic size significantly.

Some studies have suggested positive associations between dengue incidence and the *Aedes* household index and the BI, [63,64] while others have concluded that there was no significant correlation [65,66]. In our study, we found that the abundance of *Ae*. *albopictus* was almost the same for 2013 and 2014 (Fig 5), and there is no relationship between dengue incidence and the mosquito index for Guangzhou in this specific outbreak. However, on the other hand, according to our results when manipulating the climate files, the abundance of mosquitoes can affect the transmission dynamics, though does not appear to be the most important reason for the large 2014 outbreak.

There are, of course, various limitations to our analysis particularly for use in the future. For example, the whole population was considered to be susceptible in 2012 since dengue is not a common disease in Guangzhou. There were 12.70 million people at the beginning of 2012 and only 2,381 cases were reported between 2002 and 2011. In addition, there may be transmission of other serotypes in the future, only one serotype was included in the model because most of the cases have been DENV-1 in recent years. Another limitation of the study may be that the temperature dependent functions employed in the model were based on experiments which were conducted under constant temperature conditions [42,44,67]. Temperature changes from day to day as well as the diurnal temperature range can also change the transmission dynamics [68,69]. The most significant limitation, however, may be that our model does not take spatial effects into account. Further steps should be taken to develop a spatially-explicit individual based model, and to include the spatial heterogeneity and stochasticity of transmission of dengue in Guangzhou. With a stochastic model, we can learn more about the probability of local transmission, which can be combined with the outbreak scale to give us a more practical estimation of the dengue outbreak risk.

With the spread of *Ae*. *albopictus* under global warming and increasing numbers of international travelers, dengue poses additional challenges to policymakers, especially when taking into account the antibody-dependent enhancement, which can lead to increased viral replication and higher viral loads [70] when infected by another heterologous strain. A second wave outbreak with a different serotype could bring more serious manifestations of dengue fever like DHF or DSS [71]. Sustained efforts should be taken to control mosquito abundance and to prevent or limit the extent of further outbreaks.

## Supporting Information

S1 File Mathematical model description, equations and temperature- or density-dependent functions. (DOCX)

S2 File Kolmogorov plot and test result for 5 model simulation cycles. (DOCX)

S1 Table Monthly average BI and MOI for 2013 and 2014. (XLSX)

S2 Table Correlation matrix for parameters in Cycle 1 and Cycle 5. (XLSX)
