# Supplementary material for: Climate and the Timing of Imported Cases as Determinants of the Dengue Outbreak in Guangzhou, 2014: Evidence from a Mathematical Model
Source: PLoS Negl Trop Dis. 2016 Feb 10;10(2):e0004417. doi: 10.1371/journal.pntd.0004417 (PMC4749339; doi:10.1371/journal.pntd.0004417)
Supplement: S1 File — (DOCX) [file pntd.0004417.s001.docx]

**S1 Text: Mathematical model description, equations and temperature- or density-dependent functions**

**State variables**

Evu – the population of uninfected eggs

Lvu – the population of uninfected larva

Pvu – the population of uninfected pupa

Aeu – the population of uninfected emerging adults

As – the population of susceptible adults

Evi – the population of eggs infected through vertical transmission

Lvi – the population of larva infected through vertical transmission

Pvi – the population of pupa infected through vertical transmission

Aei – the population of emerging adults infected through vertical transmission

Ai – the population of infected adults

Ae – the population of exposed adults

Hs – the population of susceptible human

He – the population of exposed human

Hi – the population of infected human

Hr – the population of recovered human

**Initial values**

Evu equals to 100,000 and Hs equals to 12,700,799, which was the population for Guangzhou at the end of 2011 [1], while all others are 0. No matter what initial value we used for Evu, the Evu at the beginning of the second year keep almost the same if we keep all the other parameters at the same value, which is why we ran the model from 2012 instead of 2013.

**Differential equations**

Only females were considered in the adult stage.

$$\frac{\mathrm{dEvu}}{\mathrm{dt}}=n_{e}f_{ag}\left( As+Ae \right)+n_{e}f_{ag}\left( 1-\rho\right)Ai-{\kappa f}_{E}Evu-\mu_{E}Evu$$

$$\frac{\mathrm{dLvu}}{\mathrm{dt}}=\kappa f_{E}Evu-f_{L}Lvu-m_{L}Lvu$$

$$\frac{\mathrm{dPvu}}{\mathrm{dt}}=f_{L}Lvu-f_{P}Pvu-m_{P}Pvu$$

$$\frac{\mathrm{dAeu}}{\mathrm{dt}}={\xi e^{-\mu_{em}(1+\frac{Pvu+Pvi}{LReal})}f}_{P}Pvu-1/\gamma_{aem}Aeu-m_{A}Aeu$$

$$\frac{\mathrm{dAs}}{\mathrm{dt}}=1/\gamma_{aem}Aeu-b\alpha_{hv}\frac{Hi}{N}As-m_{A}As$$

$$\frac{\mathrm{dEvi}}{\mathrm{dt}}=\rho n_{e}f_{ag}Ai-{\kappa f}_{E}Evi-{\sigma\mu}_{E}Evi$$

$$\frac{\mathrm{dLvi}}{\mathrm{dt}}=\kappa f_{E}Evi-f_{L}Lvi-{\sigma m}_{L}Lvi$$

$$\frac{\mathrm{dPvi}}{\mathrm{dt}}=f_{L}Lvi-f_{P}Pvi-{\sigma m}_{P}Pvi$$

$$\frac{\mathrm{dAei}}{\mathrm{dt}}={\xi e^{-\mu_{em}(1+\frac{Pvu+Pvi}{LReal})}f}_{P}Pvi-{1/\gamma}_{aem}Aei-{\sigma m}_{A}Aei$$

$$\frac{\mathrm{dAi}}{\mathrm{dt}}={{1/\gamma}_{aem}Aei+f}_{exv}Ae-{\sigma m}_{A}Ai$$

$$\frac{\mathrm{dAe}}{\mathrm{dt}}={b\alpha_{hv}\frac{Hi}{N}As-f}_{exv}Ae-m_{A}Ae$$

$$\frac{\mathrm{dHs}}{\mathrm{dt}}=\alpha_{H}N-b\alpha_{vh}\frac{Ai}{N}Hs-\mu_{H}Hs$$

$$\frac{\mathrm{dHe}}{\mathrm{dt}}=b\alpha_{vh}\frac{Ai}{N}Hs-{{1/\tau}_{exh}He-\mu}_{H}He$$

$$\frac{\mathrm{dHi}}{\mathrm{dt}}={1/\tau_{exh}He-{1/\tau}_{ih}Hi-\mu}_{H}Hi$$

$$\frac{\mathrm{dHr}}{\mathrm{dt}}={{1/\tau}_{ih}Hi-\mu}_{H}Hr$$

$$N=Hs+He+Hi+Hr$$

**Water level [2]**

Water level is calculated from the following equation:

$$\omega_{Real+1}=\left\{ \begin{matrix} \omega_{Real} - EV, & \omega_{Real} + RF - EV > \omega_{max} \\ \omega_{Real}+RF - EV, & \omega_{min}< \omega_{Real}+RF - EV < \omega_{max} \\ \omega_{Real}+RF, & \omega_{Real} + RF - EV < \omega_{min} \end{matrix} \right.$$

Where

$\omega_{Real}$– daily real water level (mm)

ω_max_ – maximum water level, beyond which the potential breeding sites will overflow (mm)

ω_min_ – minimum water level which represents water containers and other standing water shielded from evaporation (mm)

EV – daily evaporation (mm)

RF – daily rainfall (mm)

**Spillover Effect [2]**

When there are heavy rains and the water levels are close to ω_max_, a fraction ω of the immature mosquito stages will be washed out from their breeding sites such as water container. Heavy rain was defined by daily cumulative precipitation greater than 50mm.

$$\omega=\omega_{0}\times\frac{1.2{\times(\frac{\omega_{Real}}{\omega_{max}})}^{20}}{1+1.2{\times(\frac{\omega_{Real}}{\omega_{max}})}^{20}}$$

**Diapause**

The eggs of *Aedes albopictus* will diapause in the winter from late October to mid-March [3]. In this period, we assumed that no eggs will hatch till the temperature and humidity are more favorable.

**Imported cases**

On day β_2013_ and β_2014_, one case was imported to Guangzhou, which was performed by adding 1 to the Hi (infected human) compartment in the model at these two days.

**Interventions**

On every Friday afternoon from October 9^th^ to early November in 2013 and from September 24^th^ to late November in 2014, ultra-low-volume adulticides were used to kill the adult stage of *Ae. albopictus*. Water containers were also emptied to remove immature mosquito stages and destroy their breeding sites. For these days, we assumed that a fraction (1-μ_a_) of adults were killed and (1-μ_i_) of immature stages and water level were removed.

Constant

| Parameter | Definition | Value | Note |
| --- | --- | --- | --- |
| κ | Diapause | 0 or 1 | 0 in October 25^th^ to March 15^th^, 1 otherwise [3] |
| μ_H_ | Mortality rate for residents in Guangzhou | 0.000035 | 1/Average life expectancy [1,4,5] |
| α_H_ | Population growth rate | 0.000081 | Estimated from population from 2010 to 2013 [1,4,5] |
| ξ | Sex ratio of *Aedes albopictus* at the emergence | 0.5 | [[6](#_ENREF_6),[7](#_ENREF_7)] |

Parameter

| Parameter | Definition |
| --- | --- |
| μ_E_ | Egg mortality rate (day^-1^) |
| θ | The ratio of minimum egg hatching rate to ideal egg hatching rate (day^-1^) |
| λ | The ratio of minimum larvae development rate to ideal larvae development rate (day^-1^) |
| ω_0_ | The maximum heavy rain washout fraction |
| ω_min_ | Minimum water level (mm) |
| ω_max_ | Maximum water level (mm) |
| π_max_ | Maximum carrying capacity for immature stages |
| γ_aem_ | Duration from emerging adults to adults (day) |
| μ_em_ | Mortality during adult emergence (day^-1^) |
| σ | The ratio of infected to uninfected immature and mature mosquito death rate |
| ρ | Vertical transmission rate, the proportion of infected eggs laid by infected mosquitoes |
| τ_exh_ | Intrinsic incubation period (day) |
| τ_ih_ | Recovery period (day) |
| α_vh_ | Transmission probability from vector to human |
| α_hv_ | Transmission probability from human to vector |
| φ | Report rate |
| β_2013_ | Time for the imported case in 2013 |
| β_2014_ | Time for the imported case in 2014 |
| μ_a_ | The survival rate for adults after intervention |
| μ_i_ | The survival rate for immature stage after intervention |

**Temperature-dependent rate**

Enzyme kinetics model based on absolute reaction rate kinetics of enzyme was used to estimate the temperature-dependent developmental rates of eggs, larva, pupa, gonotrophic cycle and extrinsic incubation rate [8].

$$r\left( T_{t} \right)=\frac{\rho(25℃)\times(T_{t}/298){\times e}^{\frac{\Delta H_{A}}{R}(\frac{1}{298}-\frac{1}{T_{t}})}}{1+e^{\frac{\Delta H_{H}}{R}(\frac{1}{T_{1/2H}}-\frac{1}{T_{t}})}}$$

Where

r(T_t_) – the development rate (hr^-1^) at temperature T (°K) on day t

T_t_ – the mean temperature (°K) on day t

∆H_A_ – the enthalpy of activation of the reaction that is catalyzed by the enzyme (cal mol^-1^)

∆H_H_ – the enthalpy change associated with high temperature inactivation of the enzyme (cal mol^-1^)

H_1/2H_ – the temperature at which half of the enzyme is inactived from high temperature

R – Universal gas constant (1.987 cal mol^-1^ deg^-1^)

Then the coefficient ρ(25℃), ∆H_A_, ∆H_H_, and H_1/2H_ were estimated from literatures with experiments conducted in Guangzhou, which typically have the development time under several different temperature.

However, mortality rate and biting rate were estimated from experiments by using quadratic or piecewise functions [9,10].

| Temperature-dependent (Fit curve with temperature) | | |
| --- | --- | --- |
| Function | Definition | Expression |
| feideal | Ideal development rate for eggs, only depend on temperature (day^-1^) | $feideal= 24\times\frac{0.00835\times(T_{t}/298){\times e}^{\frac{46701.2}{R}(\frac{1}{298}-\frac{1}{T_{t}})}}{1+e^{\frac{309796.0}{R}(\frac{1}{313.511}-\frac{1}{T_{t}})}}$ |
| mlideal | Ideal mortality rate for larva, only depend on temperature (day^-1^) | $mlideal= \left\{ \begin{matrix} 0.0000866T^{2}-0.00368T+0.0451, & T\geq12.5℃ \\ 0.5, & T<12.5 ℃ \end{matrix} \right.$ |
| flideal | Ideal development rate for larva, only depend on temperature (day^-1^) | $flideal= 24\times\frac{0.00608\times(T_{t}/298){\times e}^{\frac{51681.3}{R}(\frac{1}{298}-\frac{1}{T_{t}})}}{1+e^{\frac{186888.0}{R}(\frac{1}{313.208}-\frac{1}{T_{t}})}}$ |
| mpideal | Mortality rate for pupa (day^-1^) | $mp= \left\{ \begin{matrix} 0.01, & 12.5℃\leq T\leq35.0℃ \\ 0.5, & else \end{matrix} \right.$ |
| fp | Temperature-dependent development rate of pupae to emerging adults (day^-1^) | $fp= 24\times\frac{0.0143\times(T_{t}/298){\times e}^{\frac{44093.2}{R}(\frac{1}{298}-\frac{1}{T_{t}})}}{1+e^{\frac{100261}{R}(\frac{1}{330.058}-\frac{1}{T_{t}})}}$ |
| ma | Temperature-dependent death rate for adults (day^-1^) | $ma= \left\{ \begin{matrix} 0.000114T^{2}-0.00427T+0.0639, & T\geq15.0℃ \\ 0.5, & T<15.0 ℃ \end{matrix} \right.$ |
| fag | Temperature-dependent duration for gonotrophic cycle (day^-1^) | $fag= 24\times\frac{0.0102\times(T_{t}/298){\times e}^{\frac{60513.2}{R}(\frac{1}{298}-\frac{1}{T_{t}})}}{1+e^{\frac{705550}{R}(\frac{1}{308.352}-\frac{1}{T_{t}})}}$ |
| fexv | 1/Extrinsic incubation period (day^-1^) | $fexv= 24\times\frac{0.00333\times(T_{t}/298){\times e}^{\frac{70802.6}{R}(\frac{1}{298}-\frac{1}{T_{t}})}}{1+e^{\frac{177239}{R}(\frac{1}{448.619}-\frac{1}{T_{t}})}}$ |
| b | Biting rate (day^-1^) | Max(-0.004981T^2^+0.274T -2.94,0) |
| n_e_ | Eggs per gonotrophic cycle (per female) | Max(-0.5717T^2^+31.8313T-349.8819,0) |

**Water level dependent rate [2]**

| Water level dependent | | |
| --- | --- | --- |
| Function | Definition | Expression |
| L | The number of larva in the system | L = Lvu + Lvi |
| LReal | The carrying capacity of mosquito larvae population | L_Real_ =π_max_ *$\frac{\omega_{real}}{\omega_{max}}$ |
| fe | Real egg development rate | $fe=\left( feideal-feideal*\theta\right)*\frac{20\left( \frac{\omega_{real}}{\omega_{max}} \right)^{8}}{1+20\left( \frac{\omega_{real}}{\omega_{max}} \right)^{8}}+feideal*\theta$ |
| fl | Real larva development rate | $fl=\left( flideal-flideal*\lambda\right)*\frac{2\left( \frac{L}{L_{Real}} \right)^{-1}}{1+2\left( \frac{L}{L_{Real}} \right)^{-1}}+flideal*\lambda$ |
| ml | Real mortality for larva | ml = mlideal*(1+L/LReal) |
| F | Fraction of eggs, larvae and pupae will be washed out on each day | $F=\omega_{0}*\frac{1.2{(\frac{\omega_{real}}{\omega_{max}})}^{20}}{1+1.2{(\frac{\omega_{real}}{\omega_{max}})}^{20}}$ |

A parameter set was considered to be in the passing group, only when the number of daily new cases output by the model, calculated as *He*τ_exh_*φ*, following all of the eight conditions (also shown in Fig 3):

1. The number of daily new cases is greater than 0 and lower than 10 in at least one day between Day 600 and Day 620 (August 22^nd^ to September 11^th^, 2013, Day 1 is January 1^st^, 2012);
2. The peak of daily new cases in 2013 occurs between Day 648 and Day 668 (October 9^th^ to October 29^th^, 2013);
3. The peak amount for 2013 is greater than 10 and lower than 60;
4. The number of daily new cases is greater than 0 and lower than 10 in at least one day between Day 690 and Day 710 (November 20^th^ to December 10^th^, 2013);
5. The number of daily new cases is greater than 5 and lower than 60 in at least one day between Day 945 and Day 955 (August 2^nd^ to August 12^th^, 2014);
6. The peak of daily new cases in 2014 occurrs between Day 995 and Day 1015 (September 21^st^ to October 11^th^, 2014);
7. The peak amount for 2014 is greater than 600 and lower than 2000;
8. The number of daily new cases is greater than 5 and lower than 60 in at least one day between Day 1045 and Day 1055 (November 10^th^ and November 20^th^, 2014).

**References**

1. Statistical Bureau of Guangdong. Guangdong Statistical Yearbook 2012. Beijing: China Statistical Publishing House; 2012.

2. Karl S, Halder N, Kelso JK, Ritchie SA, Milne GJ. A spatial simulation model for dengue virus infection in urban areas. BMC Infect Dis. 2014 Aug 20; 14: 447.

3. Huang E, Wu Z. Biological characteristics and seasonal abundance of Aedes albopictus. Fujian Nong Lin Da Xue Xue Bao (Zi Ran Ke Xue Ban) 2006; 3: 246-50.

4. Statistical Bureau of Guangdong. Guangdong Statistical Yearbook 2013. Beijing: China Statistical Publishing House; 2013.

5. Statistical Bureau of Guangdong. Guangdong Statistical Yearbook 2014. Beijing: China Statistical Publishing House; 2014.

6. Liu Z, Zhang Y, Yang Y. Population dynamics of Aedes (Stegomyia) albopictus (skuse) under laboratory conditions. Kun Chong Xue Bao. 1985; 28: 274-80.

7. Delatte H, Gimonneau G, Triboire A, Fontenille D. Influence of temperature on immature development, survival, longevity, fecundity, and gonotrophic cycles of Aedes albopictus, vector of chikungunya and dengue in the Indian Ocean. J Med Entomol. 2009 Jan 1; 46(1): 33-41.

8. Sharpe PJ, DeMichele DW. Reaction kinetics of poikilotherm development. J Theor Biol. 1977 Feb 21; 64(4): 649-70.

9. Li J, Zhu G, Zhou H, Tang J, Cao J. Effect of different temperatures on development of Aedes albopictus. Zhongguo Xue Xi Chong Bing Fang Zhi Za Zhi.2015 Feb; 27(1): 1-3.

10. Zhong Z, He G. The life table of laboratory Aedes albopictus under various temperatures. Zhongshan Yi Ke Da Xue Xue Bao. 1988; 9(3): 35-9.
