# Supplementary material for: Climate and the Timing of Imported Cases as Determinants of the Dengue Outbreak in Guangzhou, 2014: Evidence from a Mathematical Model
Source: PLoS Negl Trop Dis. 2016 Feb 10;10(2):e0004417. doi: 10.1371/journal.pntd.0004417 (PMC4749339; doi:10.1371/journal.pntd.0004417)
Supplement: S2 File — (DOCX) [file pntd.0004417.s002.docx]

**S2 Text: Kolmogorov plot and test result for 5 model simulation cycles**

**Cycle 1:** The first result with a wide range for each parameter.

Passing rate: 74/410594 = 0.018%


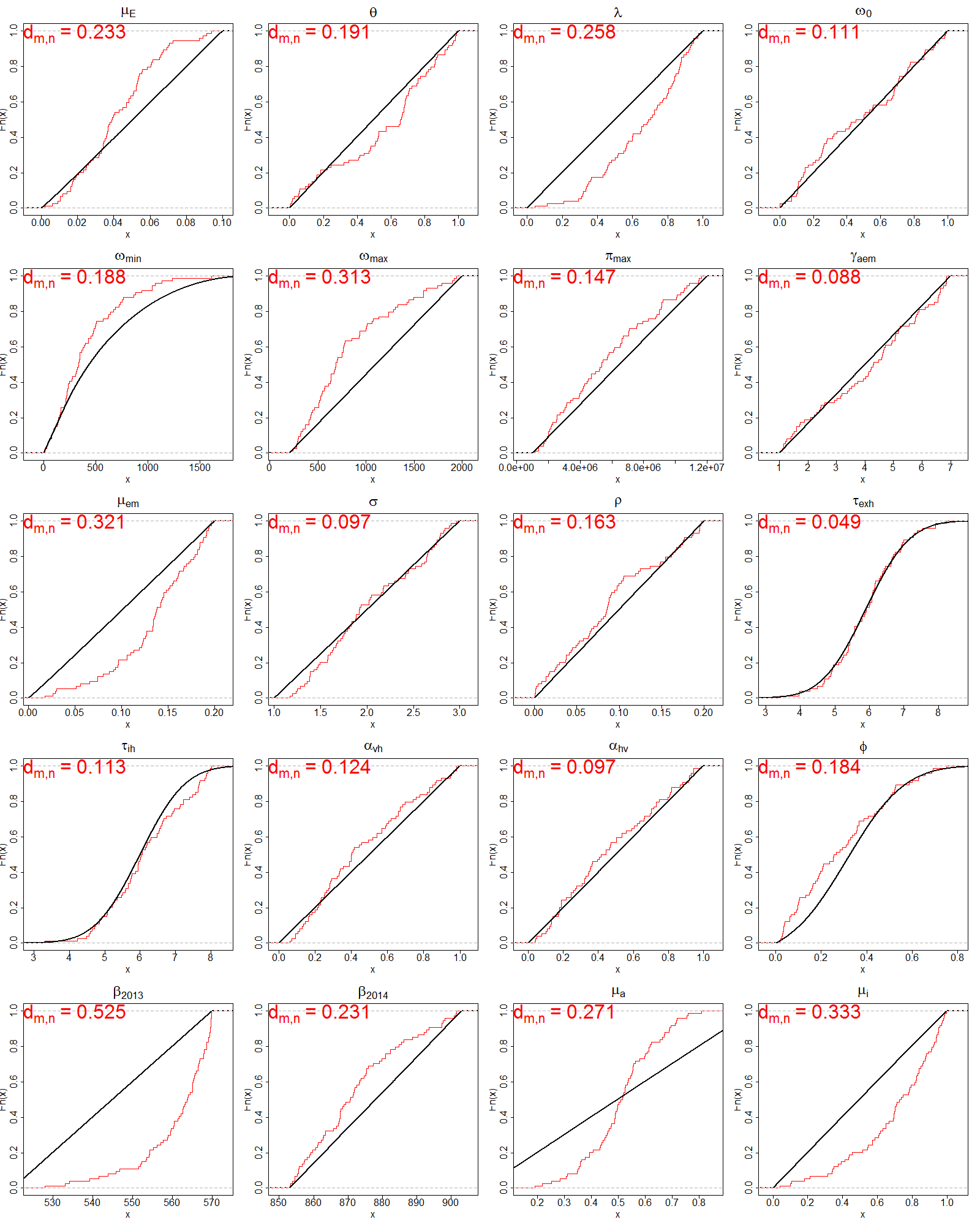


|  | PassMean | FailMean | PassStd | FailStd | dmn | pvalue |
| --- | --- | --- | --- | --- | --- | --- |
| μ_E_ | 0.042 | 0.050 | 0.021 | 0.029 | 0.233 | 0.001 |
| θ | 0.558 | 0.498 | 0.305 | 0.289 | 0.191 | 0.009 |
| λ | 0.652 | 2.035 | 0.230 | 974 | 0.258 | 0.000 |
| ω_0_ | 0.479 | 0.708 | 0.305 | 136 | 0.111 | 0.319 |
| ω_min_ | 411 | 550 | 328 | 436 | 0.188 | 0.011 |
| ω_max_ | 825 | 1100 | 468 | 519 | 0.313 | 0.000 |
| π_min_ | 5712296 | 6481347 | 3037606 | 3173687 | 0.147 | 0.083 |
| γ_aem_ | 4.124 | 4.000 | 1.838 | 1.731 | 0.088 | 0.618 |
| μ_em_ | 0.135 | 0.102 | 0.045 | 0.945 | 0.321 | 0.000 |
| σ | 2.033 | 8.658 | 0.528 | 4222 | 0.097 | 0.489 |
| ρ | 0.090 | 0.100 | 0.060 | 0.059 | 0.163 | 0.040 |
| τ_exh_ | 5.910 | 5.901 | 0.965 | 1.008 | 0.049 | 0.995 |
| τ_ih_ | 6.156 | 5.999 | 1.075 | 0.998 | 0.113 | 0.305 |
| α_vh_ | 0.464 | 0.500 | 0.268 | 0.289 | 0.124 | 0.208 |
| α_hv_ | 0.467 | 0.500 | 0.278 | 0.288 | 0.097 | 0.486 |
| φ | 0.292 | 0.337 | 0.198 | 0.178 | 0.184 | 0.013 |
| β_2013_ | 561 | 545 | 9.396 | 14.480 | 0.525 | 0.000 |
| β_2014_ | 872 | 878 | 13.579 | 14.575 | 0.231 | 0.001 |
| μ_a_ | 0.512 | 0.498 | 0.131 | 0.288 | 0.271 | 0.000 |
| μ_i_ | 0.694 | 0.497 | 0.242 | 0.288 | 0.333 | 0.000 |

**Cycle 2:** Result after using a narrow range

Passing rage: 826/172070 = 0.48%


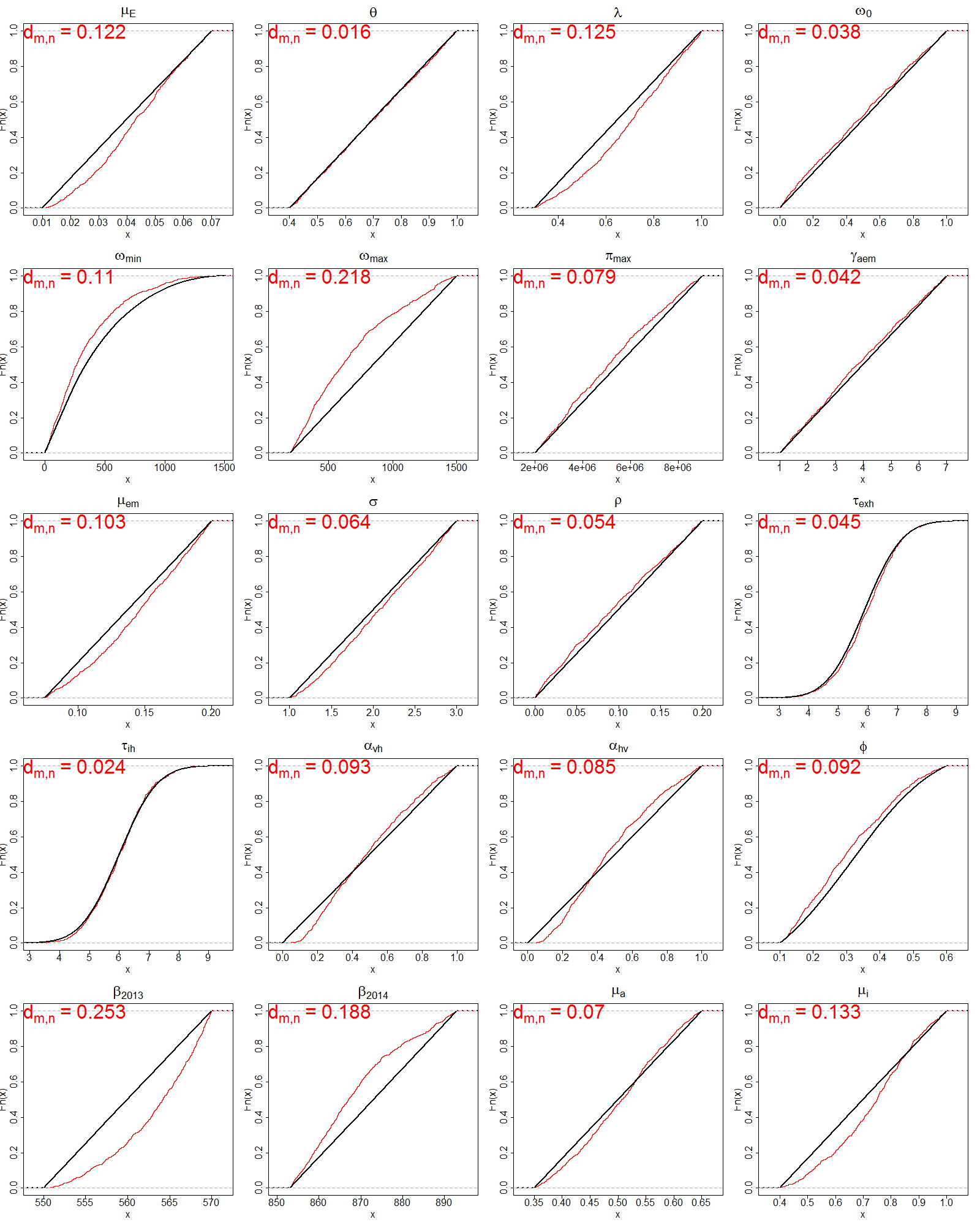


|  | PassMean | FailMean | PassStd | FailStd | dmn | pvalue |
| --- | --- | --- | --- | --- | --- | --- |
| μ_E_ | 0.043 | 0.040 | 0.015 | 0.017 | 0.122 | 0.000 |
| θ | 0.701 | 0.699 | 0.173 | 0.173 | 0.016 | 0.986 |
| λ | 0.695 | 0.649 | 0.187 | 0.202 | 0.125 | 0.000 |
| ω_0_ | 0.477 | 0.499 | 0.292 | 0.288 | 0.038 | 0.178 |
| ω_min_ | 350 | 425 | 290 | 327 | 0.110 | 0.000 |
| ω_max_ | 685 | 850 | 353 | 375 | 0.218 | 0.000 |
| π_min_ | 5206392 | 5495510 | 1975305 | 2021386 | 0.079 | 0.000 |
| γ_aem_ | 3.882 | 3.996 | 1.726 | 1.732 | 0.042 | 0.112 |
| μ_em_ | 0.144 | 0.137 | 0.034 | 0.036 | 0.103 | 0.000 |
| σ | 2.073 | 2.002 | 0.553 | 0.577 | 0.064 | 0.003 |
| ρ | 0.094 | 0.100 | 0.059 | 0.058 | 0.054 | 0.018 |
| τ_exh_ | 5.963 | 5.899 | 0.979 | 1.002 | 0.045 | 0.069 |
| τ_ih_ | 6.014 | 5.999 | 0.954 | 1.002 | 0.024 | 0.734 |
| α_vh_ | 0.499 | 0.500 | 0.249 | 0.288 | 0.093 | 0.000 |
| α_hv_ | 0.485 | 0.500 | 0.246 | 0.289 | 0.085 | 0.000 |
| φ | 0.311 | 0.334 | 0.130 | 0.130 | 0.092 | 0.000 |
| β_2013_ | 563 | 560 | 4.994 | 5.773 | 0.253 | 0.000 |
| β_2014_ | 869 | 873 | 10.884 | 11.550 | 0.188 | 0.000 |
| μ_a_ | 0.504 | 0.500 | 0.078 | 0.087 | 0.070 | 0.001 |
| μ_i_ | 0.734 | 0.700 | 0.148 | 0.173 | 0.133 | 0.000 |

**Cycle 3:** narrow down the range of the second run

Passing rate: 264/20000 = 1.320%


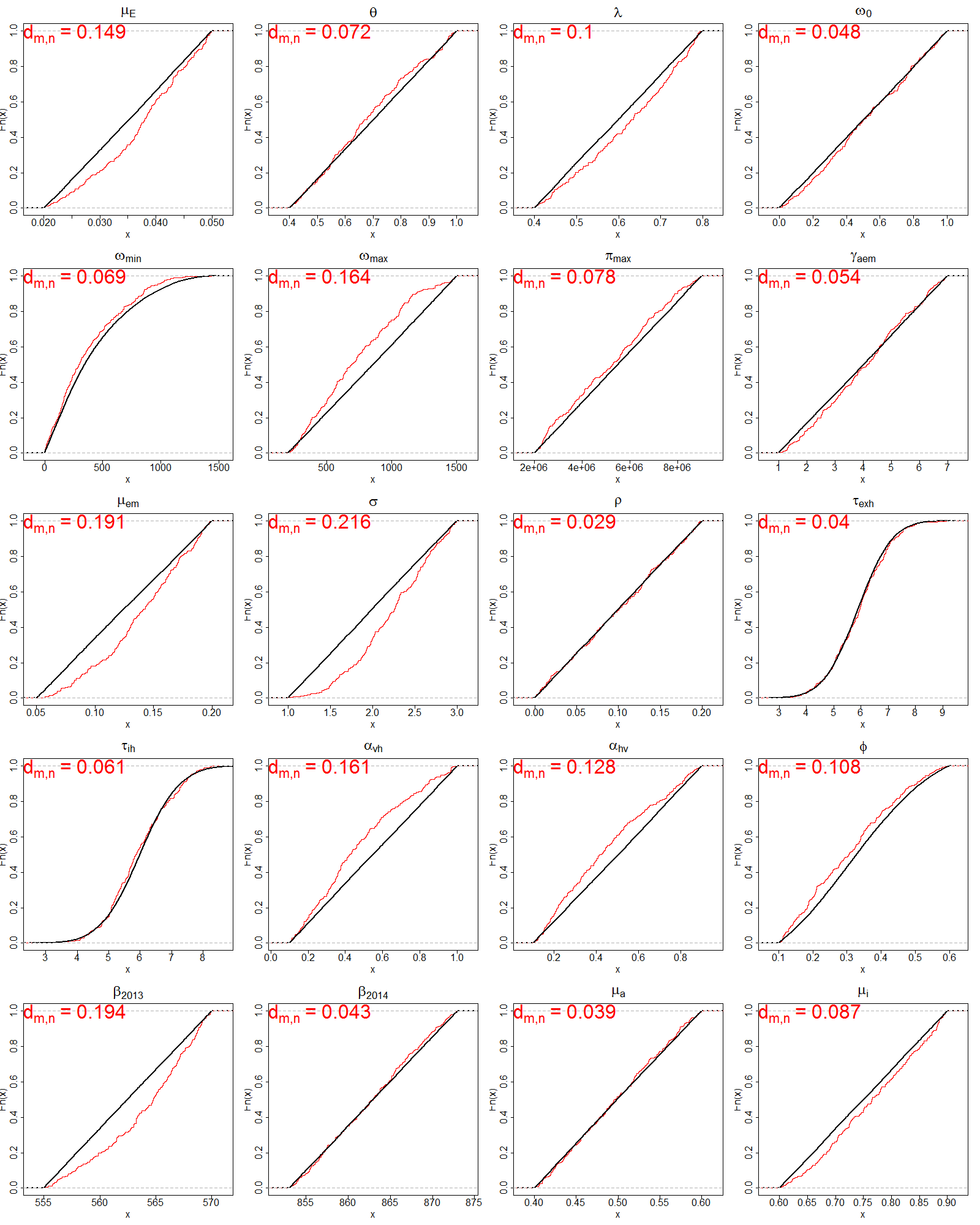


|  | PassMean | FailMean | PassStd | FailStd | dmn | pvalue |
| --- | --- | --- | --- | --- | --- | --- |
| μ_E_ | 0.037 | 0.035 | 0.008 | 0.009 | 0.149 | 0.000 |
| θ | 0.687 | 0.701 | 0.169 | 0.173 | 0.072 | 0.134 |
| λ | 0.622 | 0.599 | 0.115 | 0.116 | 0.100 | 0.011 |
| ω_0_ | 0.517 | 0.503 | 0.278 | 0.289 | 0.048 | 0.591 |
| ω_min_ | 378 | 426 | 302 | 329 | 0.069 | 0.165 |
| ω_max_ | 736 | 854 | 337 | 375 | 0.164 | 0.000 |
| π_min_ | 5214565 | 5501201 | 1994530 | 2019452 | 0.078 | 0.083 |
| γ_aem_ | 4.072 | 4.007 | 1.612 | 1.732 | 0.054 | 0.432 |
| μ_em_ | 0.139 | 0.125 | 0.037 | 0.043 | 0.191 | 0.000 |
| σ | 2.222 | 1.996 | 0.462 | 0.576 | 0.216 | 0.000 |
| ρ | 0.100 | 0.100 | 0.058 | 0.058 | 0.029 | 0.981 |
| τ_exh_ | 5.918 | 5.888 | 1.039 | 1.002 | 0.040 | 0.810 |
| τ_ih_ | 5.960 | 5.999 | 1.010 | 1.002 | 0.061 | 0.289 |
| α_vh_ | 0.471 | 0.549 | 0.243 | 0.261 | 0.161 | 0.000 |
| α_hv_ | 0.450 | 0.503 | 0.228 | 0.231 | 0.128 | 0.000 |
| φ | 0.310 | 0.333 | 0.132 | 0.130 | 0.108 | 0.004 |
| β_2013_ | 564 | 562 | 4.007 | 4.338 | 0.194 | 0.000 |
| β_2014_ | 863 | 863 | 5.516 | 5.756 | 0.043 | 0.728 |
| μ_a_ | 0.498 | 0.500 | 0.056 | 0.058 | 0.039 | 0.833 |
| μ_i_ | 0.764 | 0.750 | 0.083 | 0.087 | 0.087 | 0.038 |

**Cycle 4**: narrow down the range of the third run

Passing rate: 516/20000 = 2.580%


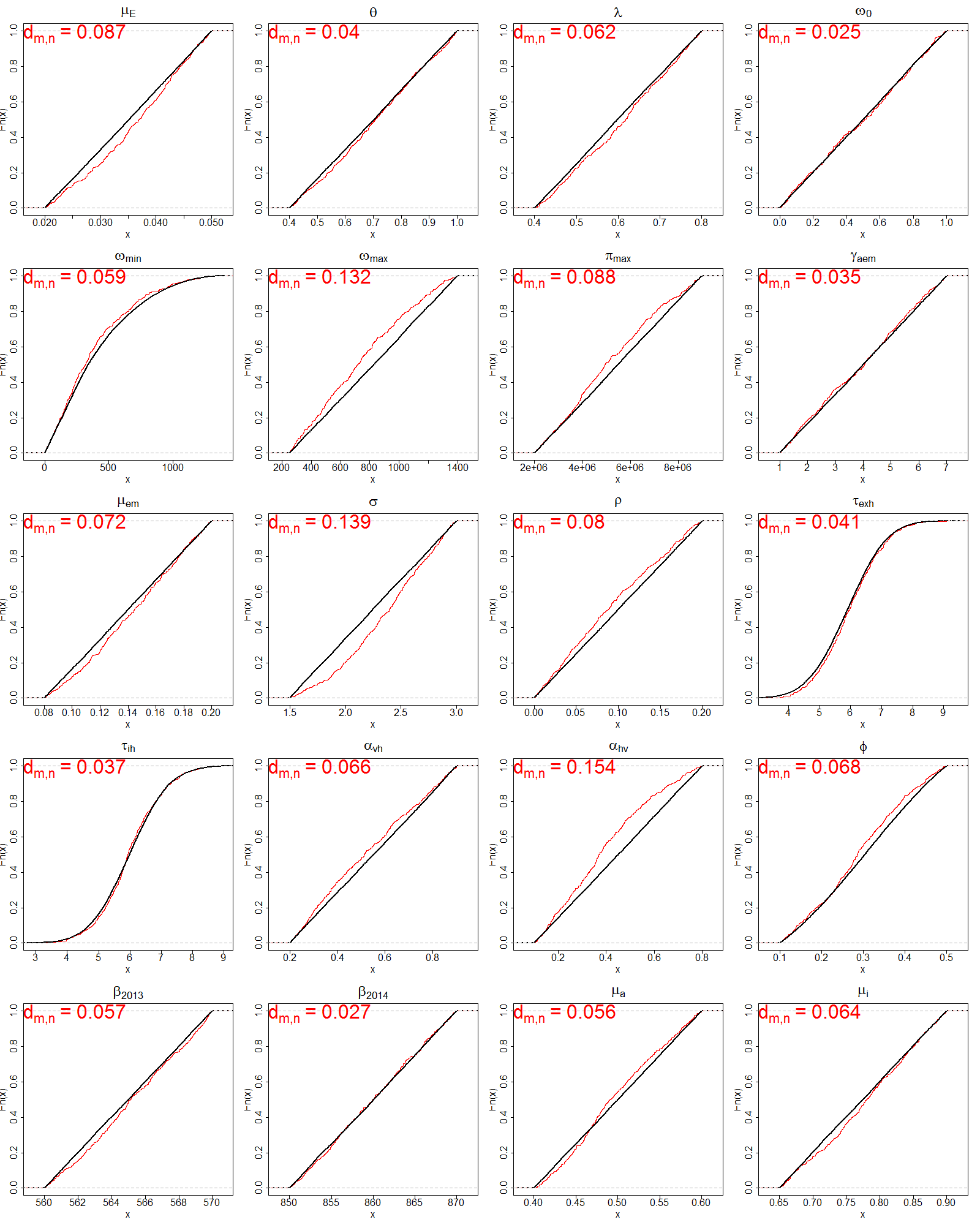


|  | PassMean | FailMean | PassStd | FailStd | dmn | pvalue |
| --- | --- | --- | --- | --- | --- | --- |
| μ_E_ | 0.036 | 0.035 | 0.008 | 0.009 | 0.087 | 0.001 |
| θ | 0.709 | 0.701 | 0.170 | 0.173 | 0.040 | 0.392 |
| λ | 0.610 | 0.600 | 0.113 | 0.115 | 0.062 | 0.043 |
| ω_0_ | 0.496 | 0.497 | 0.290 | 0.287 | 0.025 | 0.914 |
| ω_min_ | 385 | 412 | 296 | 306 | 0.059 | 0.062 |
| ω_max_ | 744 | 826 | 313 | 331 | 0.132 | 0.000 |
| π_min_ | 5199985 | 5494038 | 1931414 | 2009862 | 0.088 | 0.001 |
| γ_aem_ | 3.933 | 4.008 | 1.728 | 1.733 | 0.035 | 0.562 |
| μ_em_ | 0.144 | 0.140 | 0.033 | 0.035 | 0.072 | 0.011 |
| σ | 2.357 | 2.248 | 0.386 | 0.434 | 0.139 | 0.000 |
| ρ | 0.092 | 0.100 | 0.057 | 0.058 | 0.080 | 0.003 |
| τ_exh_ | 5.979 | 5.901 | 0.962 | 1.003 | 0.041 | 0.381 |
| τ_ih_ | 5.999 | 5.994 | 0.972 | 1.012 | 0.037 | 0.499 |
| α_vh_ | 0.527 | 0.552 | 0.205 | 0.203 | 0.066 | 0.025 |
| α_hv_ | 0.396 | 0.451 | 0.183 | 0.201 | 0.154 | 0.000 |
| φ | 0.291 | 0.303 | 0.104 | 0.108 | 0.068 | 0.020 |
| β_2013_ | 565 | 565 | 2.846 | 2.893 | 0.057 | 0.075 |
| β_2014_ | 860 | 860 | 5.646 | 5.771 | 0.027 | 0.862 |
| μ_a_ | 0.498 | 0.500 | 0.054 | 0.058 | 0.056 | 0.086 |
| μ_i_ | 0.779 | 0.774 | 0.070 | 0.072 | 0.064 | 0.034 |

**Cycle 5**: narrow down the range of the fourth run

Passing rate: 637/20000 =3.185%


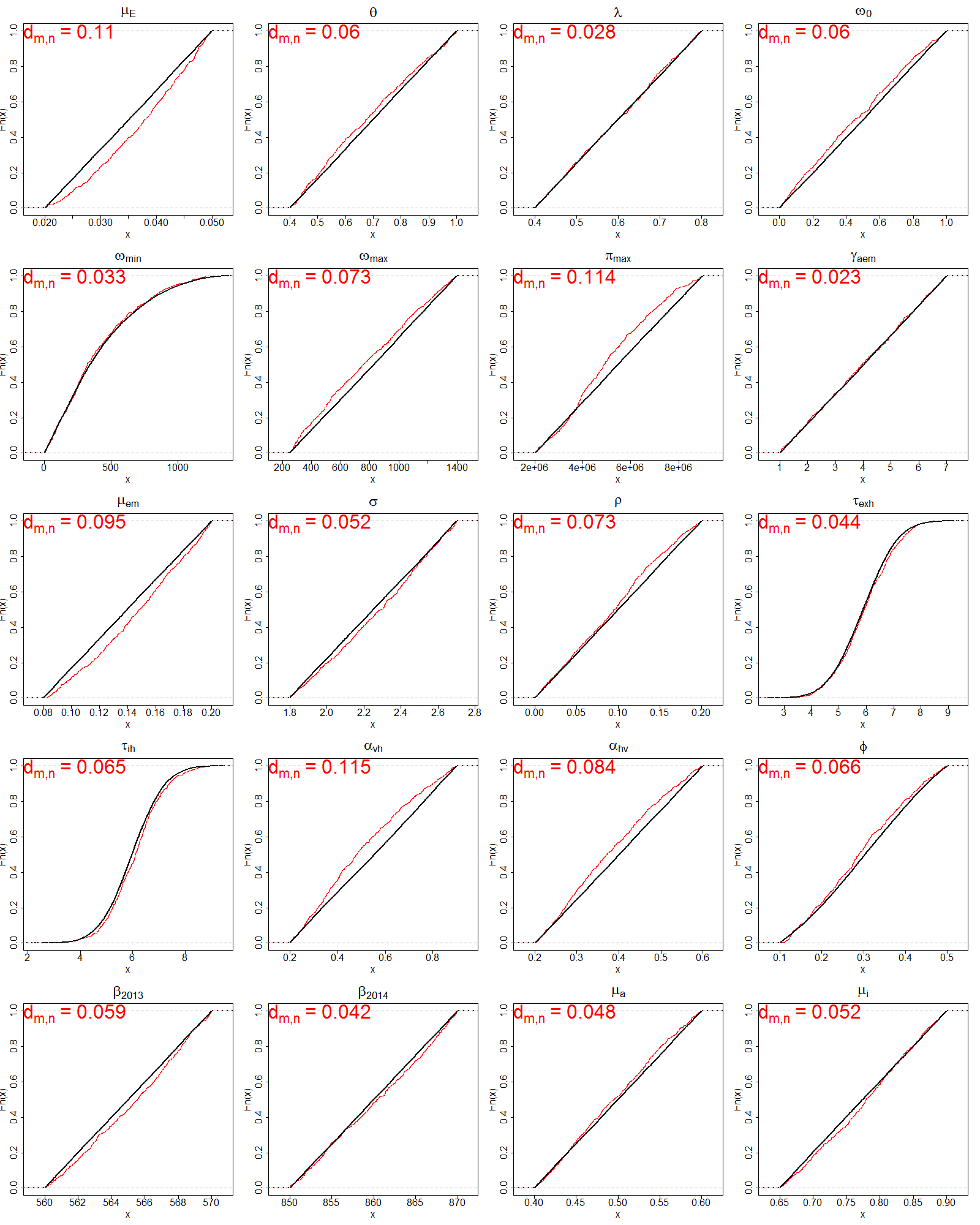


|  | PassMean | FailMean | PassStd | FailStd | dmn | pvalue |
| --- | --- | --- | --- | --- | --- | --- |
| μ_E_ | 0.037 | 0.035 | 0.008 | 0.009 | 0.110 | 0.000 |
| θ | 0.684 | 0.700 | 0.175 | 0.173 | 0.060 | 0.023 |
| λ | 0.598 | 0.600 | 0.115 | 0.116 | 0.028 | 0.733 |
| ω_0_ | 0.465 | 0.501 | 0.286 | 0.287 | 0.060 | 0.024 |
| ω_min_ | 400 | 412 | 297 | 307 | 0.033 | 0.515 |
| ω_max_ | 778 | 827 | 336 | 332 | 0.073 | 0.003 |
| π_min_ | 5104575 | 5484079 | 1813524 | 2013748 | 0.114 | 0.000 |
| γ_aem_ | 3.977 | 4.013 | 1.739 | 1.735 | 0.023 | 0.905 |
| μ_em_ | 0.147 | 0.140 | 0.033 | 0.035 | 0.095 | 0.000 |
| σ | 2.272 | 2.251 | 0.258 | 0.262 | 0.052 | 0.067 |
| ρ | 0.095 | 0.100 | 0.055 | 0.058 | 0.073 | 0.003 |
| τ_exh_ | 5.945 | 5.890 | 1.018 | 1.001 | 0.044 | 0.178 |
| τ_ih_ | 6.092 | 5.983 | 1.016 | 0.999 | 0.065 | 0.012 |
| α_vh_ | 0.507 | 0.552 | 0.194 | 0.203 | 0.115 | 0.000 |
| α_hv_ | 0.381 | 0.401 | 0.110 | 0.115 | 0.084 | 0.000 |
| φ | 0.293 | 0.303 | 0.105 | 0.108 | 0.066 | 0.009 |
| β_2013_ | 565 | 565 | 2.830 | 2.890 | 0.059 | 0.028 |
| β_2014_ | 860 | 860 | 5.899 | 5.773 | 0.042 | 0.219 |
| μ_a_ | 0.496 | 0.500 | 0.056 | 0.058 | 0.048 | 0.111 |
| μ_i_ | 0.778 | 0.775 | 0.070 | 0.072 | 0.052 | 0.068 |
